# An integrative and comparative study of pan-cancer transcriptomes reveals distinct cancer common and specific signatures

**DOI:** 10.1038/srep33398

**Published:** 2016-09-16

**Authors:** Zhen Cao, Shihua Zhang

**Affiliations:** 1 National Center for Mathematics and Interdisciplinary Sciences, Academy of Mathematics and Systems Science, Chinese Academy of Sciences, Beijing 100190, China

## Abstract

To investigate the commonalities and specificities across tumor lineages, we perform a systematic pan-cancer transcriptomic study across 6744 specimens. We find six pan-cancer subnetwork signatures which relate to cell cycle, immune response, *Sp1* regulation, collagen, muscle system and angiogenesis. Moreover, four pan-cancer subnetwork signatures demonstrate strong prognostic potential. We also characterize 16 cancer type-specific subnetwork signatures which show diverse implications to somatic mutations, somatic copy number aberrations, DNA methylation alterations and clinical outcomes. Furthermore, some of them are strongly correlated with histological or molecular subtypes, indicating their implications with tumor heterogeneity. In summary, we systematically explore the pan-cancer common and cancer type-specific gene subnetwork signatures across multiple cancers, and reveal distinct commonalities and specificities among cancers at transcriptomic level.

Cancer is a very heterogeneous disease which shows distinct diversity in genomics^1^. Hanahan and Weinberg summarized eight well-known hallmarks and two enabling characteristics of cancers which provide solid foundations of cancer biology and suggest new directions for cancer research^2^. With the rapid development of high-throughput technologies, several large-scale projects like The Cancer Genome Atlas (TCGA) and International Cancer Genome Consortium (ICGC) have been launched for about ten years to generate and profile large amounts of molecular data at the genomic, transcriptomic, proteomic and epigenomic levels^3^. Nowadays, bioinformatics communities are facing unprecedented opportunities and challenges to turn such massive cancer molecular profiling data into realistic knowledge^4^,^5^,^6^.

In this background, pan-cancer study is becoming a new and valuable paradigm to explore the comprehensive cancer molecular profiling data^7^,^8^,^9^. Hoadley *et al.* conducted a multiplatform pan-cancer analysis across twelve cancer types and found a subtype consisting of lung squamous, head and neck, and a subset of bladder cancers, which are characterized by *TP53* alterations, *TP63* amplifications, and deregulation of immune and proliferation genes^4^. Gevaert *et al.* performed a pan-cancer DNA methylation analysis on combined cancer types and got 10 clusters of patients, revealing new epigenomic similarities across malignances^10^. Yang *et al.* also employed a pan-cancer study to demonstrate universal patterns of epigenomic deregulation and distinct processes controlling genome-wide DNA hypo- and hyper-methylation across tumor lineages^11^. More recently, Andor *et al.* explored the intratumor heterogeneity using exome sequences in twelve cancer types, demonstrating its widespread existence as well as clinical implications^12^. However, how these biological factors regulate downstream gene expression is still a challenging issue^13^.

Transcriptomic data is one of the most commonly available high-throughput molecular data, playing critical roles in exploring underlying characteristics of cancer and designing new drug targets. Generally, transcriptomic regulation are heavily influenced by somatic copy number alterations (SCNA), DNA methylation alterations and other regulatory factors^11^,^14^. Moreover, transcriptomic data pinpoint to some key intrinsic molecular subtypes and have been used as one key factor for the prediction of clinical outcomes^15^,^16^. For example, Heiser *et al.* analyzed transcriptomic data of a cohort of breast cancer cell lines and revealed subtype and pathway-specific responses to anticancer compounds^17^. Liu *et al.* applied a network tool to transcriptional profiles of 917 cancer cell lines and identified 14 robust biological meaningful subnetworks associated with multiple cancer activities^18^. Zhang *et al.* built a weighted frequent gene co-expression network and found 13 cancer networks relating to several key common cancer traits and identified a set of genes involving in genome stability^19^. More recently, Biton *et al.* identified 20 independent components relating to tumor cells, tumor microenvironment and nonbiological factors in bladder cancer transcriptome using independent component analysis^20^. However, to our knowledge, there is no large-scale pan-cancer study to systematically explore the cancer common and specific gene transcriptomic subnetwork signatures across a number of cancers.

In this study, we aim to explore the commonalities across tumor lineages and shed light on cancer specificities using large-scale RNA-seq data across 16 cancer types. Strikingly, we find six pan-cancer gene subnetwork signatures, most of which relate to well-known cancer hallmarks, indicating the existence of common cancer characteristics. On the other hand, we depict significantly biological-relevant cancer type-specific subnetwork signatures which distinctly pinpoint to cancer specificity and pathology of some given cancer types.

## Result

### Overview of the pan-cancer transcriptomic analysis

We obtain the gene expression data of 6744 specimens across 16 cancer types from TCGA and preprocess the data of each cancer type with standard methods (Methods and Supplementary Table S1). These 16 cancer types include bladder urothelial carcinoma (BLCA), breast invasive carcinoma (BRCA), cholangiocarcinoma (CHOL), colon adenocarcinoma (COAD), glioblastoma multiforme (GBM), head and neck squamous cell carcinoma (HNSC), kidney chromophobe (KICH), kidney renal clear cell carcinoma (KIRC), kidney renal papillary cell carcinoma (KIRP), liver hepatocellular carcinoma (LIHC), lung adenocarcinoma (LUAD), lung squamous cell carcinoma(LUSC), prostate adenocarcinoma (PRAD), rectum adenocarcinoma (READ), thyroid carcinoma (THCA) and uterine corpus endometrial carcinoma (UCEC).

**Figure 1:**
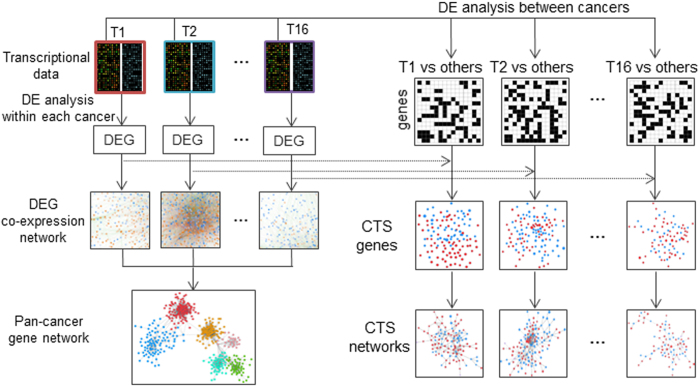
The workflow for constructing a pan-cancer gene network and cancer type-specific networks. To construct a pan-cancer gene network, we first conduct differential expression analysis between tumors and corresponding normal controls to get DEGs and then construct a DEG co-expression network for each cancer. These 16 DEG co-expression networks are then merged into a pan-cancer network which are divided into six distinct modular subnetworks using a network partition method^21^. To construct cancer type-specific subnetworks, we first conduct differential expression analysis between a given cancer and all others, and select cancer type-specific genes within DEGs relative to both normal controls and other cancer types. Then we use a web tool called geneMania^22^,^23^ to integrate known reliable interactions and choose the derived largest connected component as the cancer type-specific subnetwork. Abbreviation. T: tissue. DE: differential expression. DEG: differentially expressed genes. CTS: cancer type-specific.

**Figure 2:**
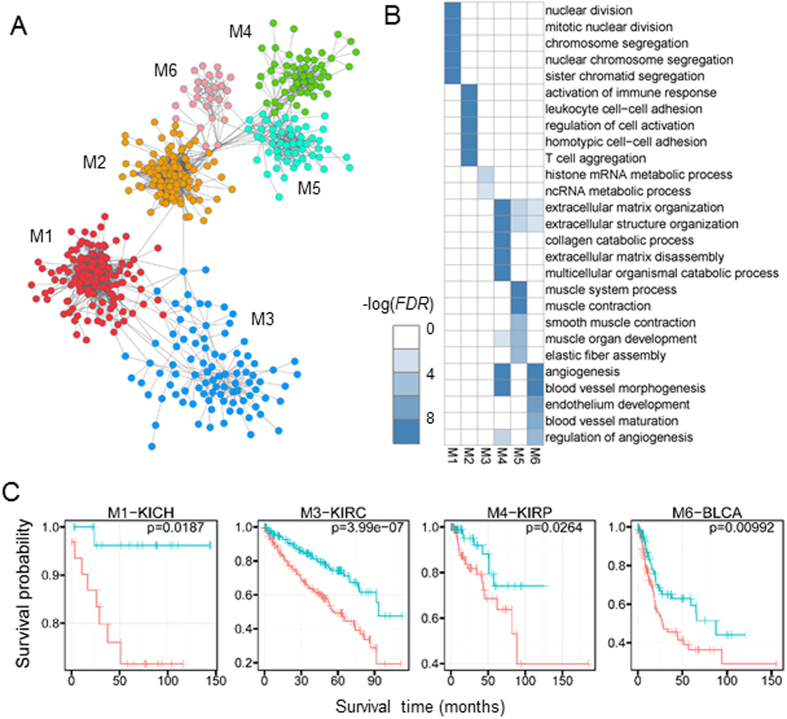
The pan-cancer network. (**A**) Topological organization of the pan-cancer network which show six modular subnetworks marked with different colours. (**B**) Enriched biological functions of pan-cancer subnetworks. Each row represents a GO BP term and each column corresponds to a pan-cancer subnetwork. For each subnetwork, *FDRs* are calculated using Fisher’s exact test and Benjamini-Hochberg correction^59^. For each subnetwork, we only show the top five significant terms. Each pixel represents a −log(*FDR*) (*FDR* ≤ 0.01). *FDRs* under 1 × 10^−10^ are changed into 1 × 10^−10^ for convenience. (**C**) Pan-cancer subnetworks relate to prognostic information. For a given cancer type and a given pan-cancer subnetwork/module, patients are divided into two groups based on the median of the ME score. The Kaplan-Meier survival curves are drawn for each group. Four representative cases are shown^65^ with *P* values calculated by log rank sum test. Each subfigure corresponds to a subnetwork and a cancer type.

We conduct a systematic and integrative pan-cancer analysis to explore pan-cancer modular subnetworks and cancer type-specific subnetworks (Fig. 1 and Supplementary Table S2). Specifically, to construct a pan-cancer network, we first determine differentially expressed genes (DEGs) by comparing expression level of tumors to normal samples and then construct a cancer type-specific DEG co-expression network for each cancer. We further select edges appearing in no less than three co-expression networks and combine all these edges and linking genes to construct a pan-cancer network. We can clearly see that the pan-cancer network shows distinct modular organization with six modular subnetworks. We then use a network partition method developed by Newman^21^ to decompose this network (Fig. 2A, Supplementary Figure S1 and Table S2). For cancer type-specific subnetworks, we conduct differential expression analysis between a given cancer and others. For each cancer, cancer type-specific genes are then selected according to differential expression analysis relative to both normal controls and other cancer types. Based on these cancer type-specific genes, we use a web tool called geneMania^22^,^23^ to integrate known reliable networks and choose the largest connected component as the targeted cancer type-specific subnetwork (or module).

### Pan-cancer modular subnetworks reveal common cancer signatures

Our pan-cancer subnetworks show distinct biological relevance to tumorigenesis and tumor progression (Fig. 2B). Our further study shows that they are all associated with some cancer hallmarks. Specifically, most of the genes in the subnetwork M1 (104/141) are involved in cell cycle. *RB* and *TP53* are two critical switches of cell cycle progression, which control whether or not a cell ought to continue its growth-and-division round. Generally, dysregulation of these cell cycle genes with defects of proteins *RB* and *TP53* will permit persistent cell proliferation of cancer cells and promote tumor progression in the long term^2^. From another perspective, alterations in cyclin-dependent kinase (CDK) activity often induce and regulate cell cycle defects in tumors^24^. Interestingly, one node *CDK1* of this subnetwork has been reported to drive the cell cycle with its partners cyclins A2 and B1^25^. Moreover, the relatively high connection (with 51 neighbor genes) in the subnetwork M1 with its explicit function further suggests its role as therapeutic target^2^,^26^.

In the whole pan-cancer network, the subnetwork M3 is highly connected with the subnetwork M1 relative to other pairs (Fig. 2A). The biological functions ‘histone mRNA and non-coding RNA metabolic process’ of this subnetwork imply its involvement in epigenomic regulation (Fig. 2B). Furthermore, we find that most genes (62/91) in the subnetwork M3 may be *cis*-regulated by *Sp1*. Many motifs are significantly enriched in the promoter regions of these genes and a number of these motifs specifically bind to protein *Sp1* (Methods and Supplementary Figure S2A). Moreover, the widespread abnormality of DNA methylation levels in promoter regions influence the binding of *Sp1* to these GC-enriched motifs and further affect the gene expressions (Supplementary Figure S2B). But there are some exceptions, which may be elucidated by cancer heterogeneity or other regulatory patterns like SCNAs. In recent studies, relationship between *Sp1* and the hallmarks of cancer has been well explored^27^, indicating the common function of the subnetwork M3 among many cancers.

Besides M1 and M3, the other four subnetworks all point to tumor stroma (Fig. 2B). Compared to M1 and M3, these four subnetworks connect more tightly to each other topologically (Fig. 2A), implying their internal relationships. Generally, M2, M4, M5, M6 are distinctly enriched with immune system, collagen, muscle system and angiogenesis related functions, respectively. Evading immune destruction (M2) and inducing angiogenesis (M6) are two well-known cancer hallmarks, which play important roles in tumor progression^2^. M4 contains most collagen genes (16/53). Collagens are the most abundant proteins in extracellular matrix and provide structural support for cells, which play contradictory but crucial roles in cancer^28^. More interestingly, M6 is related to muscle system process and many tumor patients suffer from fatigue and muscle weakness. Such symptoms are hard to treat and recent studies have started to explore their mechanisms^29^,^30^. We conjecture that subnetwok M6 may help to understand the underlying mechanisms and screen drug targets. More importantly, these subnetworks also show distinct relevance to patient survival in several cancer types (Fig. 2C) and diverse clinical outcomes such as tumor grade (Supplementary Table S3).

### Cancer type-specific gene subnetworks demonstrate tumor specificity

**Figure 3:**
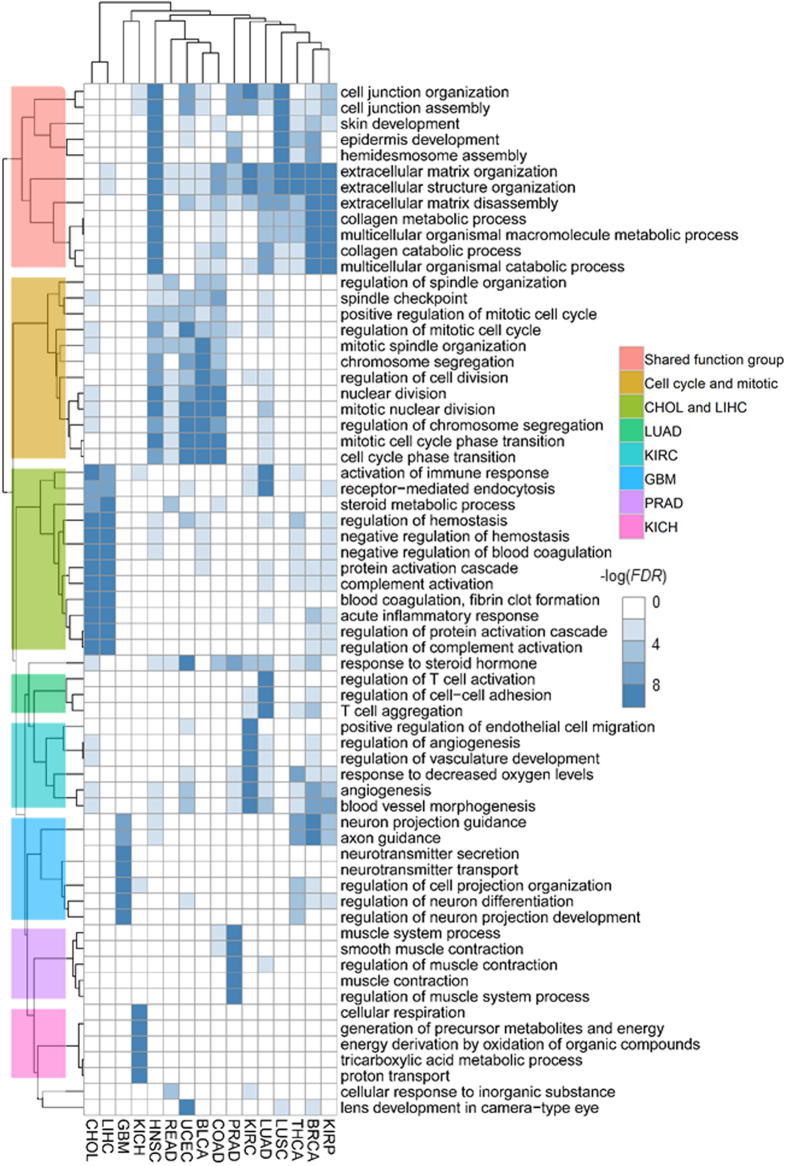
Biological functions of cancer type-specific subnetworks. Each row represents a GO BP term and each column corresponds to a cancer type-specific subnetwork. For each network, *FDRs* are calculated using Fisher’s exact test and Benjamini-Hochberg correction^59^. Each pixel represents a −log(*FDR*) (*FDR* ≤ 0.01). For each network, we only show the top five significant terms. *FDRs* under 1 × 10^−10^ are changed into 1 × 10^−10^ for convenience. Rows and columns are ordered according to the results of hierarchical clustering (Euclidean distance and average linkage). The dendrogram of BP terms is marked by eight different groups, including shared functional group, cell cycle group and six cancer type-specific ones.

We further explore the cancer type-specific modular subnetworks, which show limited number of overlaps (Supplementary Figure S3). As expected, several cancer type-specific subnetworks (CHOL, LIHC, GBM and KICH) show distinct functional relevance, indicting their cancer-specificities (Fig. 3). Specifically, the CHOL and LIHC subnetworks show similar functional relevance to blood coagulation and inflammatory response, which is consistent with previous observations^31^, implying significant implications of liver hepatocellular carcinoma with hepatitis^32^. The GBM subnetwork is related to neural system, which shows distinct difference from others and reflects distinct tissue specificity. Although the functional terms of the PRAD subnetwork look similar to the pan-cancer subnetwork M5, it still shows functional specificity of PRAD relating to known phenomenon. For example, androgen dependent treatments usually reduce testosterone levels and cause loss of muscle for PRAD cohort. The KIRC subnetwork is enriched in angiogenesis and cell migration which is consistent with that KIRC is a typical metabolic disease. In summary, these observations suggest that the defined subnetworks do point to tumor initiation and metastasis^33^,^34^.

On the other hand, most of such subnetworks are enriched in the part of the shared functional groups including extracellular matrix, cell junction related functions and so on. These functions help to provide organized environment for cells and to orchestrate cells into higher level organizations as well^35^,^36^. The deregulation of these functions remind us of the metastasizing features across different cancer types while the specific signatures of these subnetworks imply the distinct mechanisms of metastases^36^. Apart from the shared functional group, the BRCA, KIRP and THCA subnetworks also have the functions of angiogenesis and neuron guidance, relating to cancer cell proliferation^2^. Moreover, five cancer subnetworks including that of HNSC, READ, UCEC, BLCA and COAD are noted as cell cycle and mitotic group which have no distinct functional specificity (Fig. 3) with an exception of the UCEC subnetwork relating to response to steroid hormone.

In the following, we further explore the potential genomic and clinical relevance to demonstrate their underlying implications with tumor mechanisms. We choose the BRCA subnetwork to study its implications with breast cancer subtypes, which may give some valid insights into the tumor heterogeneity. We further use the KIRP and THCA subnetworks to illustrate their relevance to SCNA and mutations with potential oncogenic connections.

### BRCA-specific gene subnetwork relates to the basal-like subtype

**Figure 4:**
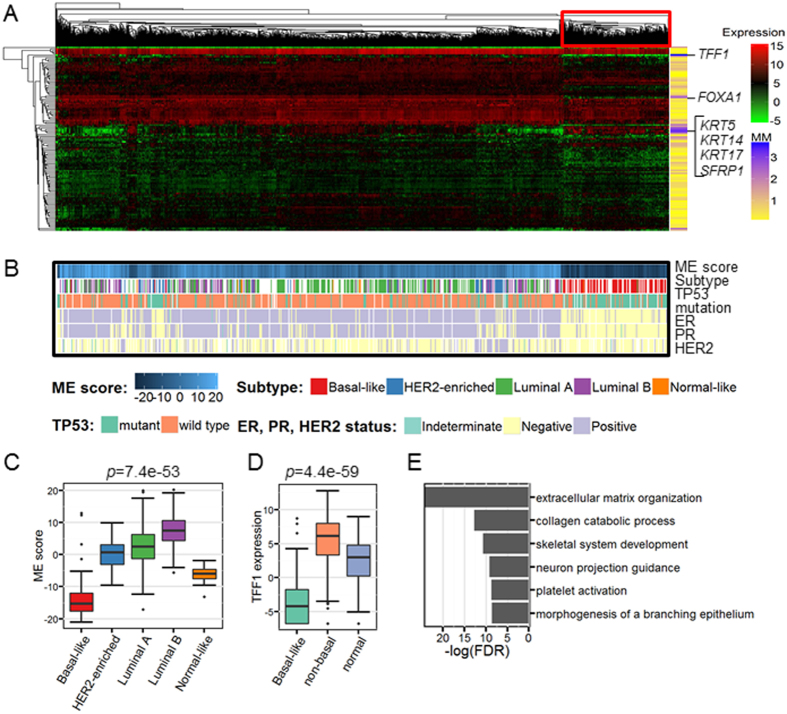
BRCA subnetwork is relevant to the basal-like subtype significantly. (**A**) Gene expression heatmap of the BRCA subnetwork (module) genes. The genes (rows) and samples (columns) are ordered according to the results of hierarchical clustering (Euclidean distance and average linkage). Those most contributing genes include three basal like markers namely *KRT5*, *KRT14* and *KRT17* and a famous prognostic marker *TFF1*. Module memberships (MM, normalized factor loadings) are indicated along the rows. (**B**) Important markers distinguish high ME score basal-like patients from others. ME scores, subtype information, *ER* status, *PR* status, *HER2* status and *TP53* mutation status are shown. ‘Equivocal’ of *HER2* status is deemed as missing values. All missing values are in white. Patients are in the same order as in the expression heatmap. (**C**) Distribution of the ME scores in terms of intrinsic subtypes. *P* value is calculated by Kruskal-Wallis test. (**D**) Distribution of the *TFF1* gene expression (TMM normalization data) in terms of the basal-subtype and others. Luminal A, luminal B, *HER2* enriched and normal like subtypes are merged as “non-basal” group. *P* value is calculated by Kruskal-Wallis test. (**E**) Functional enrichment analysis show BRCA subnetwork is related to cell proliferation. *FDR* is calculated using Fisher’s exact test and Benjamini-Hochberg correction^59^. For box plots, the bottom, top, and middle bands of the boxes indicate the 25^th^, 75^th^, and 50^th^ percentiles, respectively. Whiskers extend to the most extreme data points no more than 1.5 interquartile range from the box. *FDR* (or p value) obtained with the Kruskal-Wallis test are provided at the top of the boxplots.

We find that the BRCA-specific gene subnetwork (BRCA subnetwork) is strongly associated with the basal-like breast cancer (Fig. 4). The top four contributing genes of it include three well-known basal biomarkers *KRT5*, *KRT14* and *KRT17* (Fig. 4A)^15^,^20^. In basal-like subtype, the 5^th^ contributing gene *SFRP1* (Fig. 4A) is reported to have significantly lower DNA methylation levels and higher expression compared to luminal and *HER2*-enriched subtypes^37^,^38^. The expression of the 7^th^ contributing gene *FOXA1* (Fig. 4A) has been used to predict basal-like subtype in the PAM50 method^15^. As a result, our subnetwork genes distinguish a fraction of patients distinctly (using the hierarchical clustering with Euclidean distance and average linkage) which are likely to suffer from basal-like tumors, which has been confirmed by the known subtype information (Fig. 4A,B)^39^. We can clearly see that the basal-like patients are significantly clustered together while the patients of other subtypes are mixed chaotically (Fig. 4B). Moreover, as we expected that the putative basal-like patients (marked by a red box in Fig. 4A) tend to be *ER*, *PR* and *HER2* negative ones and they have high frequency of *TP53* mutations (Fig. 4B, Supplementary Figure S4A and S4B), which are all typical characteristics of basal-like tumors^39^,^40^. More interestingly, module eigengene (ME) score can solely distinguish these patients as well (Fig. 4B). Basal-like patients tend to have extremely low ME scores, which is quite different from other subtypes (Fig. 4C). This observation partly explains the subnetwork specificity since the basal-like tumors are quite different from luminal and *HER2*-enriched subtypes in both clinical outcomes and molecular signatures^4^,^15^.

We consider that the most contributing genes may reveal pathological mechanisms due to their strong association between this subnetwork and the basal-like subtype. The top contributing gene *TFF1* is indeed relevant to multiple breast cancer activities^41^,^42^. For example, *TFF1* is a tumor suppressor gene in gastric cancer and the deficiency in *TFF1* promotes tumorigenesis in MCF-7 cell which is a luminal subtype breast cancer cell line^43^,^44^. However, the relationship between *TFF1* and basal-like subtype still remains unclear. We can see that *TFF1* gene tend to be high-expressed in breast cancer according to differential expression analysis, but it is significantly low-expressed when restricted to basal-like subtype tumors (Fig. 4D and Supplementary Figure S4C). Dysregulation of this gene may influence the remaining genes of this subnetwork, and further accelerates cell differentiation in basal-like tumors (Fig. 4E).

### KIRP-specific subnetwork captures core SCNA characteristics

**Figure 5:**
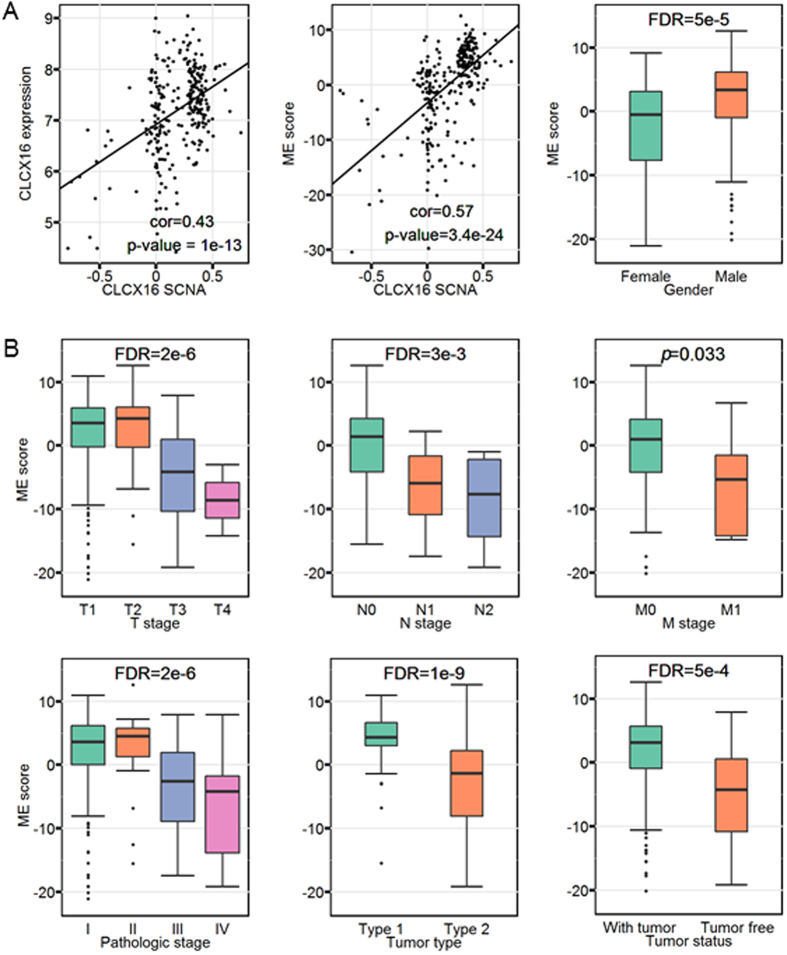
KIRP subnetwork is relevant to SCNA significantly. (**A**) KIRP subnetwork (module) relates to the core SCNA. Left: scatter plot of *CLCX16* SCNA versus *CLCX16* gene expression. Each point represents a patient. The regression line in the panel is calculated by least squares. Pearson’s correlation coefficient between *CLCX16* SCNAs and *CLCX16* gene expression and respective *p* value is shown at the bottom of the panel. Middle: scatter plot of *CLCX16* SCNAs versus ME scores. Right: distribution of the ME scores in terms of gender. (**B**) The association of KIRP subnetwork with clinical information. Distribution of the ME scores in terms of AJCC (pTNM) T stage, N stage, M stage, pathologic tumor stage, tumor status and tumor type. For box plots, the bottom, top, and middle bands of the boxes indicate the 25^th^, 75^th^, and 50^th^ percentiles, respectively. Whiskers extend to the most extreme data points no more than 1.5 interquartile range from the box. *FDR* (or *p* value) for the Kruskal-Wallis test are provided at the top of the boxplots^59^.

We find that the KIRP specific subnetwork captures core SCNA characteristics and connects these genomic alterations to downstream clinical outcomes. Previous studies have shown that malignant renal papillary cell carcinoma are marked by the trisomy of chromosomes 7, 16, 17 and the loss of Y chromosome^45^. For KIRP, the SCNAs of seven genes including *CXCL16*, *PLCD3* locating on chromosome 17 and *CLDN3, FZD1, MET, ITGB8*, *TFPI2* locating on chromosome 7 are significantly relevant to their gene expressions. Moreover, the SCNAs of these seven genes are highly correlated with ME scores (Fig. 5A and Supplementary Figure S5), indicting the potential impact of SCNAs to the KIRP-specific subnetwork. The factor loadings of these genes are relatively high, meaning that they indeed contribute a large part to the ME. Although we have no copy number data for sex chromosomes, we still see that the ME scores shows significant difference in terms of gender (Fig. 5A).

On the other hand, the SCNA characterized KIRP specific network also demonstrate strong relevance to multiple clinical outcomes (Fig. 5B), which is not biased by gender (Supplementary Table S4). Generally, the ME scores becomes lower as tumor progresses. We also notice that the ME scores in terms of AJCC (pTNM) M stage show marginal significance (Fig. 5B), which may be affected by the small sample size of given metastases status. Moreover, this subnetwork is also relevant to the tumor status as that tumor patients tend to have lower ME scores than tumor-free patients (Fig. 5B). For histological subtype, type two patients are reported to have relatively worse prognostic characteristics than type one patients and we observe that type two patients have lower ME scores than those of type one patients (Fig. 5B)^46^. These results indicate that the low ME scores of this subnetwork relate to relatively poor prognosis.

### Cancer specific-subnetworks relate to somatic mutations

**Figure 6:**
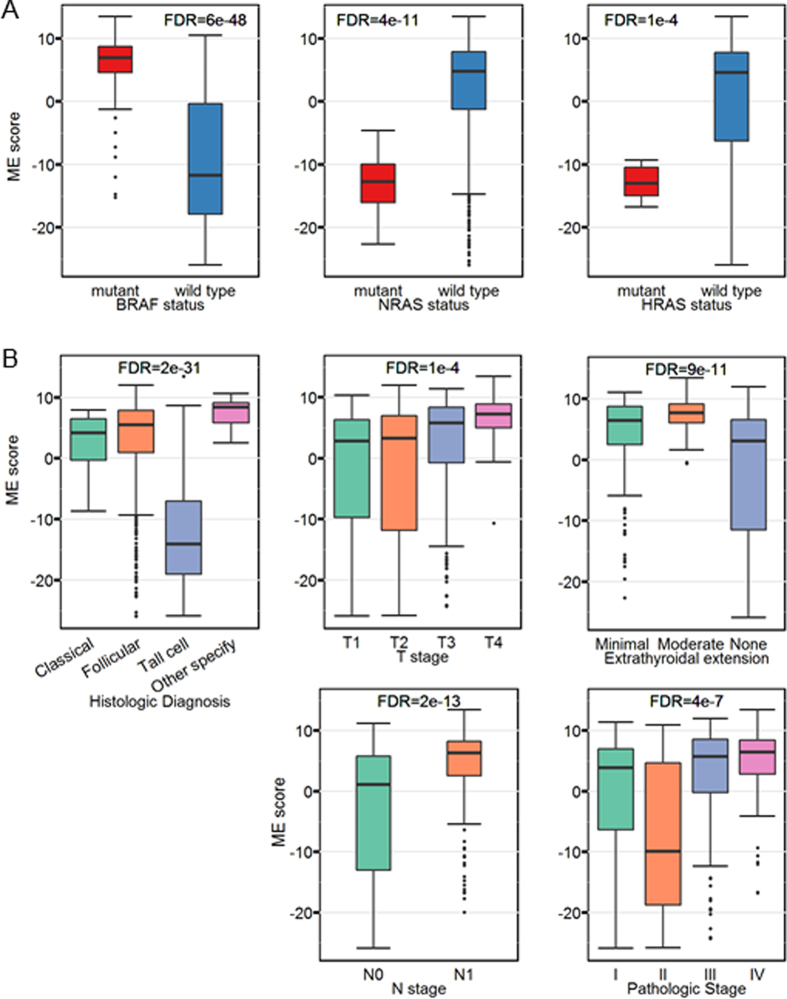
The association of THCA subnetwork with *BRAF* pathway. (**A**) THCA subnetwork (module) is associated with *RAF-RAS* mutation. Distribution of the ME scores in terms of *BRAF*, *NRAS* and *HRAS* mutation status. (**B**) The association of THCA subnetwork with clinical information. Distribution of the ME scores in terms of AJCC (pTNM) T stage, extrathyroidal extention (this partition of patients is based on T stage), N stage, and pathologic tumor stage. For box plots, the bottom, top, and middle bands of the boxes indicate the 25^th^, 75^th^, and 50^th^ percentiles, respectively. Whiskers extend to the most extreme data points no more than 1.5 interquartile range from the box. *FDR* (or *p* value) for the Kruskal-Wallis test are provided at the top of the boxplots^59^.

We find that the ME scores of the THCA-specific subnetwork are strongly associated with the mutation status of *BRAF*, *NRAS* and *HRAS*, which have relatively high mutation frequency (Fig. 6A)^47^,^48^. Those genes are at the upstream of RAS-RAF-MEK-MAP kinase signaling pathway and have been shown to play critical roles in carcinogenesis of thyroid^48^. Moreover, we observe the same strong association between these somatic mutations and histologic diagnosis in TCGA patients as well (Supplementary Figure S6A)^49^. The subnetwork is heavily influenced by the upstream signal in RAS-RAF-MEK-MAP kinase signaling pathway and have an effect on histological types because it is extremely relevant to histologic diagnosis (Fig. 6B). Although the mechanism is not totally understood due to the complexity of somatic mutations, the expression pattern of this subnetwork can provide complementary diagnostic information. Not surprisingly, we also find that this subnetwork relates to several typical clinical outcomes including T stage, extrathyroidal extension, N stage and pathologic stage (Fig. 6B).

As to the BLCA-specific subnetwork, previous studies show that it reflects the behavior of *Ta* pathway of bladder tumor progression. This subnetwork is associated with *FGFR3* mutations (Supplementary Figure S6B), which is a key marker of *Ta* pathway and therapeutic target of bladder cancer^50^. We also notice that this subnetwork is related to T stage, pathologic stage, tumor grade and histological subtype (Supplementary Figure S6B). A recent study revealed a biological component relating to both *Ta* pathway and carcinoma *in situ* pathway, of which one biomarker is early *TP53* mutation^20^. Different from that, *TP53* mutation status is not significantly relevant to the BLCA-specific subnetwork, indicating *Ta* pathway of bladder cancer reflect a cancer type-specific characteristic.

## Discussion

Integrating large-scale genomics data such as the transcriptomic data of multiple cancers to study pan-cancer and cancer-specific characteristics is an urgent and valuable paradigm for cancer biology. In this study, we find six pan-cancer subnetwork signatures associated with distinct common cancer mechanisms including cell cycle, *SP1* regulations, immune response, extracellular matrix organization, muscle system process and angiogenesis. These subnetworks provide distinct prognostic characteristics, indicating their roles as potential prognostic biomarkers. We also find 16 cancer type-specific subnetworks which demonstrate strong implications to somatic mutations, SCNAs, DNA methylation alterations and clinical outcomes in some specific cancers. These subnetworks profile the distinct specificities of cancer transcriptomes, which are often missed by non-pan-cancer studies. Not surprisingly, different cancer-specific subnetworks show very diverse implications to mutation status, SCNAs and others. Furthermore, some cancer-specific subnetworks connect upstream DNA damage to clinical outcomes, reflecting their critical roles in pathogenesis.

Our pan-cancer subnetworks reflect significant common characteristics across different cancers. Not surprisingly, they are relevant to multiple cancer hallmarks in various ways^2^. We also observe similar results in some recent pan-cancer transcriptomic analysis^19^,^20^. Strikingly, four subnetworks M2, M4, M5 and M6 pinpoint to tumor stroma, which is quite different from that of cell lines^18^. Tumor microenvironment is related to multiple cancer activities and there are many open questions in this field. Therefore, network biology offers new directions to reveal the complexity of tumor microenvironment.

Cancer type-specific subnetworks are involved in a great diversity of regulatory factors. The preference of these subnetworks relating to different regulatory factors reflects the complexity of oncogenic mechanisms in some sense. With the deepening of understanding of cancer, the nosogenesis is not only restricted to somatic mutations but also to SCNAs, some epigenomic deregulations and so on^51^,^52^. The KIRP and THCA subnetworks are two good examples (Figs 5 and 6), along with many other strong correlations to be explained. From this perspective, large-scale transcriptomic exploration will be a valuable tool for diverse implicating factors underlying transcriptome.

Cancer type-specific subnetworks also reflect the inherent similarities among diverse sets of cancers. For example, it is hard to distinguish COAD from READ using only genomic data^53^. In this study, their cancer type-specific subnetworks do also show the most overlaps (Supplementary Figure S3). We also observe similar phenomenon for CHOL and LIHC where their subnetworks share many genes (Supplementary Figure S3). In addition, BLCA, HNSC and LUSC subnetworks show significant overlaps, indicating their squamous cell features^4^. On the other hand, very few overlaps between KIRC, KIRP and KICH subnetworks indicating distinct underlying pathogenesis for these three types of kidney cancers (Fig. 3 and Supplementary Figure S3).

It is still very hard to deal with the heterogeneity of cancers. We observe multiple peaks of gene expressions (data not shown). For example, *TFF1* gene is up-regulated in the whole breast cancer cohort but down-regulated in the basal-like subtype (Fig. 4D and Supplementary Figure S4C). The multimodality of gene expression reflects the heterogeneity of cancers. However, multimodal distribution is different from normal distribution but may be mixture of multiple normal distributions, which is inappropriate for standard or modified t-test (e.g. LIMMA^54^). This is another reason why we use stringent thresholds in differential expression analysis (absolute fold change ≥2, *FDR* ≤ 0.001). Such expression patterns contain abundant biological meanings but the current analysis may ignore these. Multimodal distributions of gene expression levels potentially reflect the subtype information and diverse mechanisms of the upstream regulations or the downstream feedbacks. Using statistical methods like linear regression may help to screen out the immediate causals of multimodal distributions. Taken in this sense, it is urgent and valuable to make full use of these multimodal distributions in future studies and some useful strategies have been used to explore the DNA methylation in cancers^10^.

Apart from histological classifications, molecular subtypes based on various types of data are also studied and several solid molecular subtypes based on various types of data like SCNAs and DNA methylations have been characterized^4^,^10^,^15^,^55^. For a given cancer type, if we divide patients into subtypes in advance, the whole study here can be similarly applied. This will be beneficial to learn the heterogeneity and pathology of cancers. On the other hand, our study focuses on tumor cohorts with a give number of normal controls. This limits our study to a handful of cancer types which can be addressed by collecting more data from other resources in the future.

## Materials and Methods

### Materials and preprocessing

We download level 3 IlluminaHiSeq RNA-seq v2 gene expression data from The Cancer Genome Atlas (http://cancergenome.nih.gov/) and Broad Institute (http://gdac.broadinstitute.org/) on March 24, 2015. We treat organ-specific control samples and normal samples of matched tumors equally as normal samples for further differential gene analysis. We only take cancer types with at least five normal samples for further analysis. Finally, we obtain a total of 6744 specimens from 16 cancer types including BLCA, BRCA, CHOL, COAD, GBM, HNSC, KICH, KIRC, KIRP, LIHC, LUAD, LUSC, PRAD, READ, THCA, UCEC (Supplementary Table S1).

For a given cancer type, we use all cancer samples and normal controls and normalize them using the trimmed mean of M-values (TMM) normalization method^56^. We calculate the counts per million (CPM) of normalized data and then log2 transform them into the standard format. An average count of 0.5 is added to each observation to avoid taking log of zero. We only use the TMM normalized data for the following differential expression analysis and determine the differential expressed genes (DEG) (see below). Besides TMM normalization, we adopt upper quartile (UQ) normalization method^57^ to the combined count matrix of 6080 cancer samples (Supplementary Material and Supplementary Figure S7) and calculate logarithmic CPM values as aforementioned.

We also download IlluminaGA RNA-seq v2 gene expression data of COAD, READ and UCEC (Supplementary Table S1). We apply UQ normalization and calculate CPM values as aforementioned^57^. We use these IlluminaGA data to correlate subnetworks with mutation status (see below).

We download the mutation annotation files (MAF) of all 16 cancer types and the output of mutsig2cv which gives if a gene is significantly mutated or not from Broad Institute on July 24, 2015 (Supplementary Table S1)^47^. Multiple samples of one patient are combined to obtain the mutation data of this patient. In this study, we combine the mutations of different types together. Accordingly, a gene has two status namely ‘mutant’ and ‘wild type’ for each patient. For each cancer type, we take genes with *q*-value less than 0.1 calculated by mutsig2cv for further analysis^47^.

We download SCNA data from Broad Institute and TCGA GAF version 2.1 from http://hgwdev.cse.ucsc.edu/~cline/GAF2.1 on June 22, 2015 (Supplementary Table S1). We first map SCNA values to genes for each sample. If a gene is fully contained in a segment in terms of GAF 2.1, we assign the mean of that segment to the gene. Otherwise, we consider the values of those genes are missing. For each cancer type, genes with more than 50% missing values among all samples are deleted. Then we impute the missing values using the average values of their 10 nearest neighbors^58^. Different normal samples of the same patient are averaged since the normal state of chromosome is stable. We exclude patients with more than one tumor sample or those without any normal sample. Finally, for each patient, we take SCNA values of tumor sample minus that of normal sample as final SCNA values.

We download level 3 Illumina 450K Infinium methylation data from TCGA and annotation file for the chip from http://hgdownload.cse.ucsc.edu on June 22, 2015 (Supplementary Table S1). We treat the sequence from 1500bp upstream of transcription start site to the first exon as the promoter region of a gene. In the annotation file of Illumina 450K Infinium array, each probe is assigned to one or more part of the genes namely ‘TSS1500’, ‘TSS200’, ‘5’UTR’, ‘first exon’, ‘body’ and ‘3’UTR’. If the probe is mapped to any of the ‘TSS1500’, ‘TSS200’, ‘5’UTR’, ‘first exon’ of a gene, we consider it locates in the promoter region of this gene. Note we only need DNA methylation levels of gene promoter regions for further analysis. Then for each sample and each gene, β values of all remained probes mapped to that gene are averaged as the DNA methylation level. For each cancer type, genes with more than 50% missing values among all samples are deleted. Then we impute the missing values using the average values of their 10 nearest neighbors^58^. We exclude patients with more than one tumor sample.

We download tab separated ‘.patient’ files of 16 cancer types from TCGA Pan-cancer pages (https://www.synapse.org/#!Synapse:syn300013/files/). For each cancer type, clinical descriptions with more than 50% missing values are excluded. We delete some less important descriptions (e.g. form_completion_day). We merge AJCC TNM staging information (e.g. N1a and N1b are merged as N1). We use ‘death_days_to’, ‘last_contact_days_to’ and ‘vital status’ to construct survival information for survival analysis.

## Methods

### Determine differentially expressed genes (DEGs) relative to normal samples

For each cancer, we use LIMMA to detect DEGs relative to normal samples^54^ with the TMM normalized data as input. We first remove low expressed genes (more than 50% of samples have CPM < 10) and denote the remaining genes as high expressed ones. We apply LIMMA to obtain DEGs with absolute fold change ≥2 and FDR ≤ 0.001 within each cancer type (Supplementary Material and Supplementary Figure S8)^59^.

### Determine DEGs relative to other cancer types

Given a cancer type, we employ the same differential expression analysis procedure for discovering DEGs relative to other cancers using the UQ normalized data of those high expressed ones as input. We compare gene expressions to other cancers for each gene using two-sided t-test with different variances. Then we get a list of *p*-values for each gene (about 9000 genes relative to 15 cancer types) and corrected all these *p*-values (about 9000 × 15) using Bonferroni correction. We determine the significant genes with corrected *p*-value ≤ 0.001 and absolute fold change ≥2.

### Normalization of the data: choices of TMM and UQ

We perform two similar but different normalization methods under different hypotheses and use those under two situations. One is the trimmed mean of m-values (TMM) normalization^56^, which is a sophisticated method based on the hypothesis that most genes are not differentially expressed. This method is reported to have a good performance for the downstream analysis, especially for detecting differentially expressed genes^60^. The other is upper quartile (UQ) normalization^57^. TMM normalization may suffer from tumor heterogeneity and large amount of samples here. The hypothesis of UQ normalization is a little weak. The choice is based on experimental design, hypothesis and intuition as explained below. All normalization steps are performed using edgeR version 3.0.10^61^. The two methods look similar within each cancer (Supplementary Figure S7A). However, it has a strong bias when we combine 6080 cancer samples together (Supplementary Figure S7B).

In differential expression analysis (relative to normal samples), we only need to know which genes are differentially expressed compared to normal samples. The highly expressed genes (more than 50% of samples have CPM ≥ 10) determined by these two methods almost have no difference (Supplementary Figure S8). However, in CHOL and GBM, the DEGs determined by TMM normalization are significantly less than that of UQ normalization (Supplementary Figure S8). CHOL has the least tumor samples and GBM only has 5 organ-specific control samples. As a result, it is more likely to get some false discoveries in CHOL and GBM and the use of TMM normalization may help to control this situation. Accordingly, we choose TMM normalization for differential expression analysis within a cancer type (Supplementary Figure S8)^60^.

We do not use normal samples when we compare the gene expressions between a given cancer and others. The hypothesis of TMM normalization is too strong when we combine 6080 cancer samples together^56^. So we use UQ normalization instead and calculate CPM values, which is almost equivalent to normalized level 3 RNA-seq v2 data in TCGA.

### A pan-cancer network and its modular subnetworks

For each cancer type, we construct a DEG co-expression network using the Pearson’s correlation coefficients (PCC) between genes based on UQ normalization data^62^. We only keep the top 0.5% positive and 0.5% negative PCC as links and delete nodes without any connection to others. We combine links appearing in more than three cancers to construct a pan-cancer network and find its largest connected component shows distinct modular structure (Fig. 2A). We adopt the ‘leading eigenvector’ method developed by Newman^21^ to partition this network. This method tries to find densely connected subnetworks in a network by calculating the leading non-negative eigenvector of its modularity matrix. Finally, we get six pan-cancer modular subnetworks except a few exceptional nodes (Supplementary Material and Supplementary Figures S1, S9 and S10).

### Cancer type-specific subnetworks

Given a cancer type, we focus on DEGs relative to normal samples and define the specificity of a gene-cancer pair as the number of cancers that this gene are differentially expressed between this given cancer and others. The genes whose specificities are no less than a given threshold are further used to construct the cancer-specific subnetwork for each cancer using the geneMania tool (Supplementary Material and Supplementary Figures S11 and S12)^22^,^23^.

### Functional enrichment analysis

We adopt gProfiler^63^ to perform functional enrichment analysis for each subnetwork based on GO biological process (BP) terms. For subnetwork M3, we also pay attention to transcription binding sites (TBFs) retrieved from TRANSFAC database through a prediction pipeline by gProfiler^63^,^64^. We calculate statistical significance *p*-value using Fisher’s exact test and Benjamini-Hochberg correction^59^.

### Functional analysis of the subnetworks

For each subnetwork and all tumor samples of a given cancer, we perform principal component analysis on covariance matrix of gene expressions. The first principle component (PC), termed as module eigengene (ME) score, reflects the activity of the factor represented by the component across the samples. The elements of factor loading, termed as module membership (MM), are the Pearson’s correlations of genes with the ME score. It reflects how important a gene contributes to this ME score.

Mutation status of each gene defines groups of patients. We compare the distributions of ME scores in terms of mutation groups (mutant or wild type) using two-sided Mann-Whitney U tests. All *p*-values of a given ME are corrected by Benjamini-Hochberg correction^59^.

For SCNA data, we calculate the Pearson’s correlations between ME scores and SCNAs to check their implications. To further explain the results, we also calculate the Pearson’s correlations between gene expression levels and their corresponding SCNA levels (e.g., Fig. 5A).

We calculate the Pearson’s correlations between ME scores and DNA methylation levels of the promoter regions to check their implications. We also calculate the Pearson’s correlations between gene expression levels and their corresponding DNA methylation levels. For some differentially expressed genes, we also compare their DNA methylation levels with the mean of normal samples to explain the gene expression pattern (e.g., Supplementary Figure 3B).

In each cancer type, we compare the distributions of ME score to predefined clinical groups of patients using Kruskal-Wallis tests and two-sided Mann-Whitney U test if needed. For survival analysis, patients are divided into two groups based on the median value of ME scores. Survival curves of these two groups of patients are estimated by the Kaplan-Meier method^65^ with statistical significance calculated using Log-rank test.

## Additional Information

**How to cite this article**: Cao, Z. and Zhang, S. An integrative and comparative study of pan-cancer transcriptomes reveals distinct cancer common and specific signatures. *Sci. Rep.* **6**, 33398; doi: 10.1038/srep33398 (2016).

## Supplementary Material

Supplementary Information
