# Supplementary material for: An integrative and comparative study of pan-cancer transcriptomes reveals distinct cancer common and specific signatures
Source: Sci Rep. 2016 Sep 16;6:33398. doi: 10.1038/srep33398 (PMC5025752; doi:10.1038/srep33398)
Supplement: Supplementary Information [file srep33398-s1.pdf]

Supplementary Information for

An integrative and comparative study of pan-cancer transcriptomes reveals distinct cancer common and specific signatures

Zhen Cao, Shihua Zhang*

National Center for Mathematics and Interdisciplinary Sciences, Academy of Mathematics and Systems Science, Chinese Academy of Sciences, Beijing 100190, China

*To whom correspondence should be addressed. Tel/Fax: +86 01 82541360; Email: zsh@amss.ac.cn.

Supplementary methods

Supplementary tables' caption

Table S1

Table S2

Table S3

Table S4

Supplementary figures

Figure S1

Figure S2

Figure S3

Figure S4

Figure S5

Figure S6

Figure S7

Figure S8

Figure S9

Figure S10

Figure S11

Figure S12

Figure S13

Figure S14

References

Supplementary methods

Description of the gene expression data

IlluminaHiSeq or IlluminaGa RNA-seq v2 data are the two main types of RNA-seq data. There exists serious batch effects between these two types of data, as showed in the 19 COAD samples sequenced on both IlluminaHiSeq and IlluminaGa (data not shown). Moreover, the IlluminaHiseq data can solely meet out requirement. Thus, we don't use the lluminaGA data to reduce the batch effects.

Another concern is about the limited number of normal samples for some cancer types. Insufficient normal samples will greatly influence the statistic power in differential expression analysis. Although organ-specific control samples and tumor-matched normal samples have some differences, we treat them equally to add the number of normal samples. Finally, we choose those cohort with at least five normal samples for further analysis.

We note two tips about the gene expression data. First, some patients have more than one normal sample. We select one of them as the represented one when doing correlation analysis (mutation, SCNA, DNA methylation) and survival analysis. Second, we use IlluminaGA data of COAD, READ and UCEC to correlate mutation status with gene expression. These two things have no effects on the differential gene expression analysis and network construction.

Pan-cancer network construction and partition

We first construct a DEG co-expression network for each cancer using the UQ normalized expression data of tumor samples. The weights of links are absolute Pearson's correlation coefficients (PCC) between genes. After doing Bonferroni correction, we only keep the significant correlations as links (corrected p-value≤0.001). In addition, we treat the positive and negative links separately due to the nature of our data. We keep the top 0.5% positive and 0.5% negative links to cut many 'non-cancer specific' high corrections. We delete nodes without any connection to others. Finally, we get 16 differentially DEG co-expression networks.

The next step is to extract the shared or common part of these 16 networks. Many links appear in ***n*** networks (***n***=1, 2, 3…14, 15, 16). Links appearing in no less than 3 cancer networks are considered as significant ones. Then, an important thing is to check the signs of those significant links since positive and negative correlations represent different biological meanings. We find that all significant links show the same sign in all different networks. We merge these links as well as linked genes to construct a shared co-expression network. We consider its largest connected component as the pan-cancer network for further analysis.

We further adopt a classical spectral decomposition method [1] to extract the modular subnetworks. After deleting a few exceptional genes due to spectral decomposition, we

obtain six pan-cancer subnetworks (Supplementary Figure S1).

Stability of pan-cancer subnetworks

There are two parameters to construct the pan-cancer network: the top ***x***% links are kept in DEG co-expression network and the links occurring in more than *n* networks are kept. We determine ***n*** first. The main purpose is to study whether these highly frequent occurrence of these links are real or just by chance. Within each network, we shuffle the positions of the nodes to maintain the topological structure of the network and count the number of links occurring in *n* networks (***n***= 2, 3…14, 15, 16). We repeat this procedure 100 times. The false discovery rate is defined as mean count of random networks divided by the count of real networks (Supplementary Figure S9). We note that ***n***=1 is a special case. When ***n***=1, it is equivalent to combine all DEG co-expression networks. We finally choose ***n***=3 (FDR=0.0034).

As to the top ***x***% links kept in differentially expressed gene network, it is not easy and may has a strong effect on the final result. We test other 4 distinct values (0.1%, 1%, 1.5%, 2%) to compare their results with that of 0.5%. Parameters *n* are 2, 3, 4, 4 to maintain both the FDR (5.1%, 0.64%, 0.95%, 0.059%) and size of the shared network. We also use the optimal modularity and delete the scattered genes. The results show extremely strong similarities (Supplementary Figure S10). This implies that the majority of the shared network is very stable and the ***x***%=0.5% is reasonable.

Integration of known networks based on geneMania

We conduct this analysis for each cancer type separately. The inputs are gene lists. Given a cancer type, we first choose genes which are differentially expressed compared to normal controls. Then we consider the number a gene differentially expressed to other cancer types as a measurement of specificity for this gene-cancer pair. We use this parameter (denote as ***S***, ***S***=1, 2, 3, …, 13, 14, 15) to select genes. We use the web toll geneMania [2] with known pathway interactions and physical interactions to construct the cancer type-specific networks. The number of genes to be predicted from our gene list (denoted as ***N***) is another parameter. The output is the largest connected component of the resulted networks. We first fix ***N***=5 and set ***S*** for each cancer type as 8 (BLCA), 9 (BRCA), 9 (CHOL), 9 (COAD), 14 (GBM), 9 (HNSC), 14 (KICH), 12 (KIRC), 9 (KIRP), 15 (LIHC), 9 (LUAD), 10 (LUSC), 9 (PRAD), 9 (READ), 10 (THCA), 9 (UCEC).

Stability of the cancer type-specific modules

We test different ***S*** (ranging from 8 to 15) and ***N*** (equals to 2, 5, 10, 15). We only take the largest connected component whose sizes are larger than 20 for further analysis. The size of output is quite straightforward (Supplementary Figure S11). It depends heavily on ***S*** rather than ***N***. But when ***N*** and ***S*** become big at the same time, the proportion of the predicted genes in output is large (Supplementary Figure S12). Finally, ***N*** is set to be 5.

When ***S*** is big, the size of some resulted modules are small (usually less than 20 genes) and it is not robust for the downstream analysis (e.g., functional gene enrichment analysis

or principle component analysis). We ask for the size of the resulted modules to be larger than 80 genes for robustness and increase ***S*** as much as possible for each cancer type.

Supplementary tables

Table S1. The TCGA specimens used in this study. This table is provided as an Excel file.

Table S2. The node genes of the pan-cancer and cancer type-specific subnetworks in this study. Official gene symbols and corresponding Entrez ID are shown in two columns, respectively. This table is provided as an Excel file.

Table S3. The associations of pan-cancer subnetworks and clinical information. For each cancer and each subnetwork, the ME scores are calculated. FDR values for Kruskal-Wallis test are corrected by Benjamini and Hochberg correction [3]. *FDR* less than 0.05 are in bold. This table is provided as an Excel file.

Table S4. The associations of gender and clinical information in KIRP. *P*-values are calculated by Chi-squared tests. This table is provided as an Excel file.

Supplementary figures

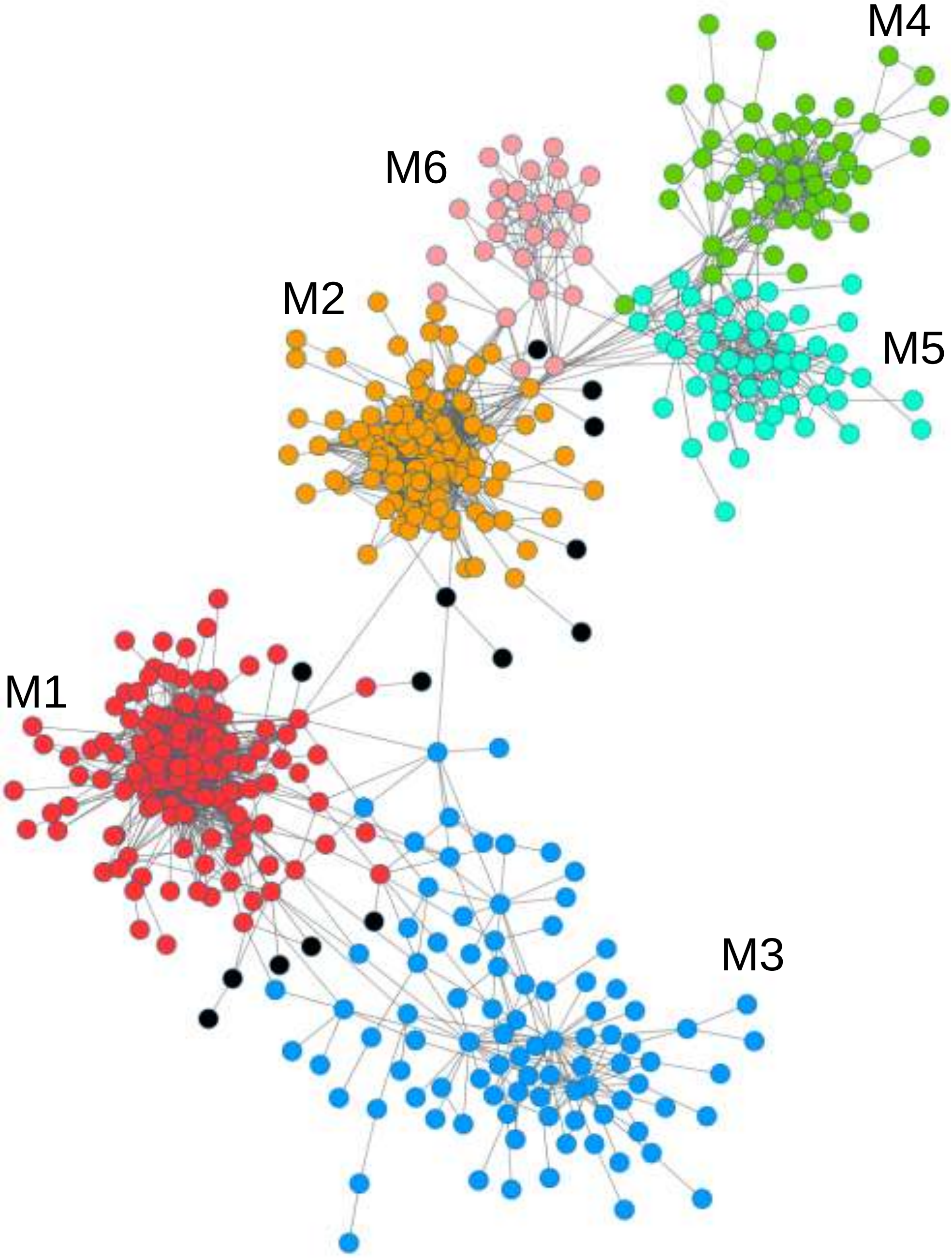

Figure S1. Topological organization of the pan-cancer networks and its six modular subnetworks indicated in different colours. Black nodes are scattered genes due to spectral decomposition.

A

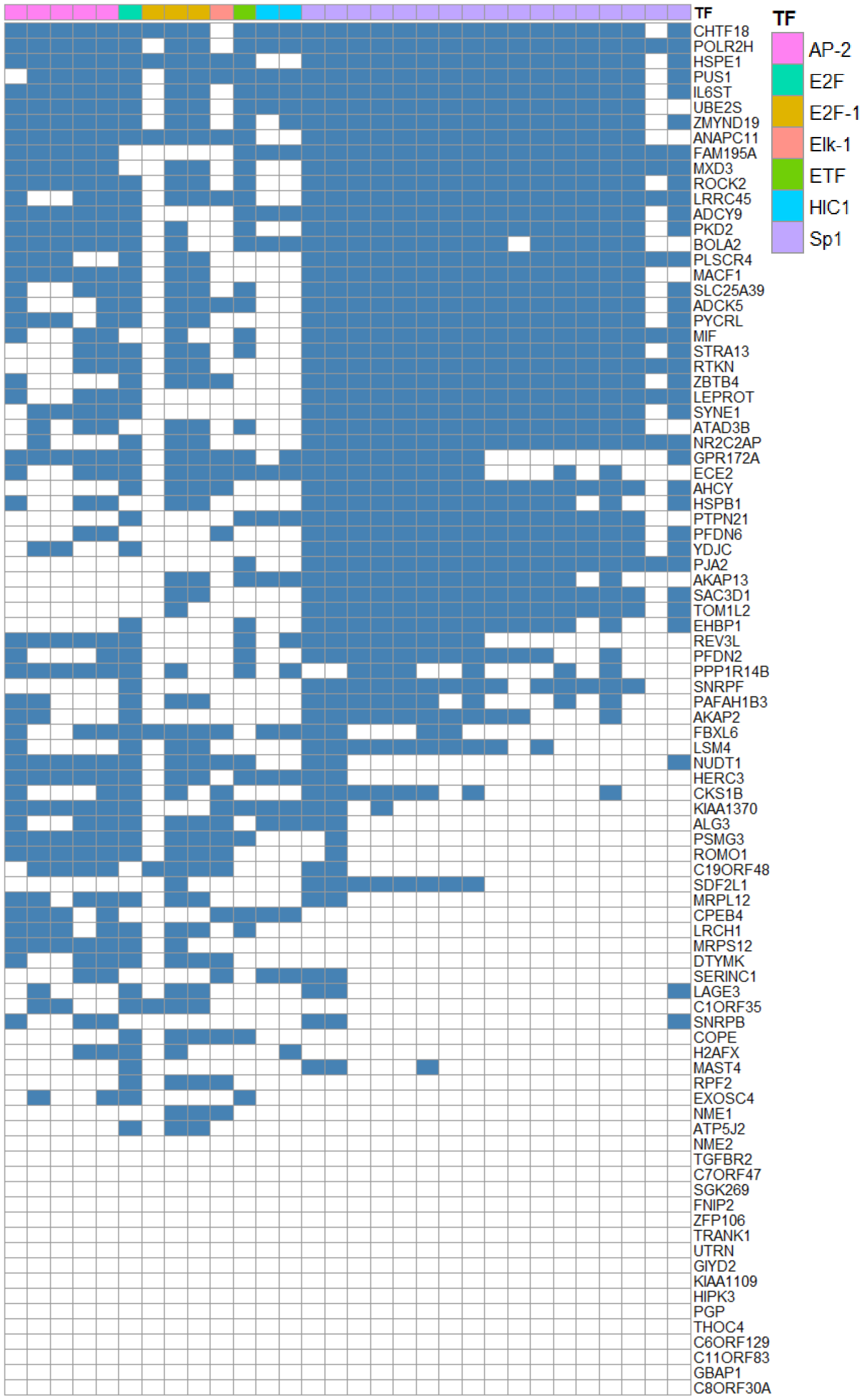
TF
CHTF18
POLR2H
HSPE1
PUS1
IL6ST
UBE2S
ZMYND19
ANAPC11
FAM195A
MXD3
ROCK2
LRRC45
ADCY9
PKD2
BOLA2
PLSCR4
MACF1
SLC25A39
ADCK5
PYCRL
MIF
STRA13
RTKN
ZBTB4
LEPROT
SYNE1
ATAD3B
NR2C2AP
GPR172A
ECE2
AHCY
HSPB1
PTPN21
PFDN6
YDJC
PJA2
AKAP13
SAC3D1
TOM1L2
EHBP1
REV3L
PFDN2
PPP1R14B
SNRPF
PAFAH1B3
AKAP2
FBXL6
LSM4
NUDT1
HERC3
CKS1B
KIAA1370
ALG3
PSMG3
ROMO1
C19ORF48
SDF2L1
MRPL12
CPEB4
LRCH1
MRPS12
DTYMK
SERINC1
LAGE3
C1ORF35
SNRPB
COPE
H2AFX
MAST4
RPF2
EXOSC4
NME1
ATP5J2
NME2
TGFBR2
C7ORF47
SGK269
FNIP2
ZFP106
TRANK1
UTRN
GIYD2
KIAA1109
HIPK3
PGP
THOC4
C6ORF129
C11ORF83
GBAP1
C8ORF30A
TF
AP-2
E2F
E2F-1
Elk-1
ETF
HIC1
Sp1

B

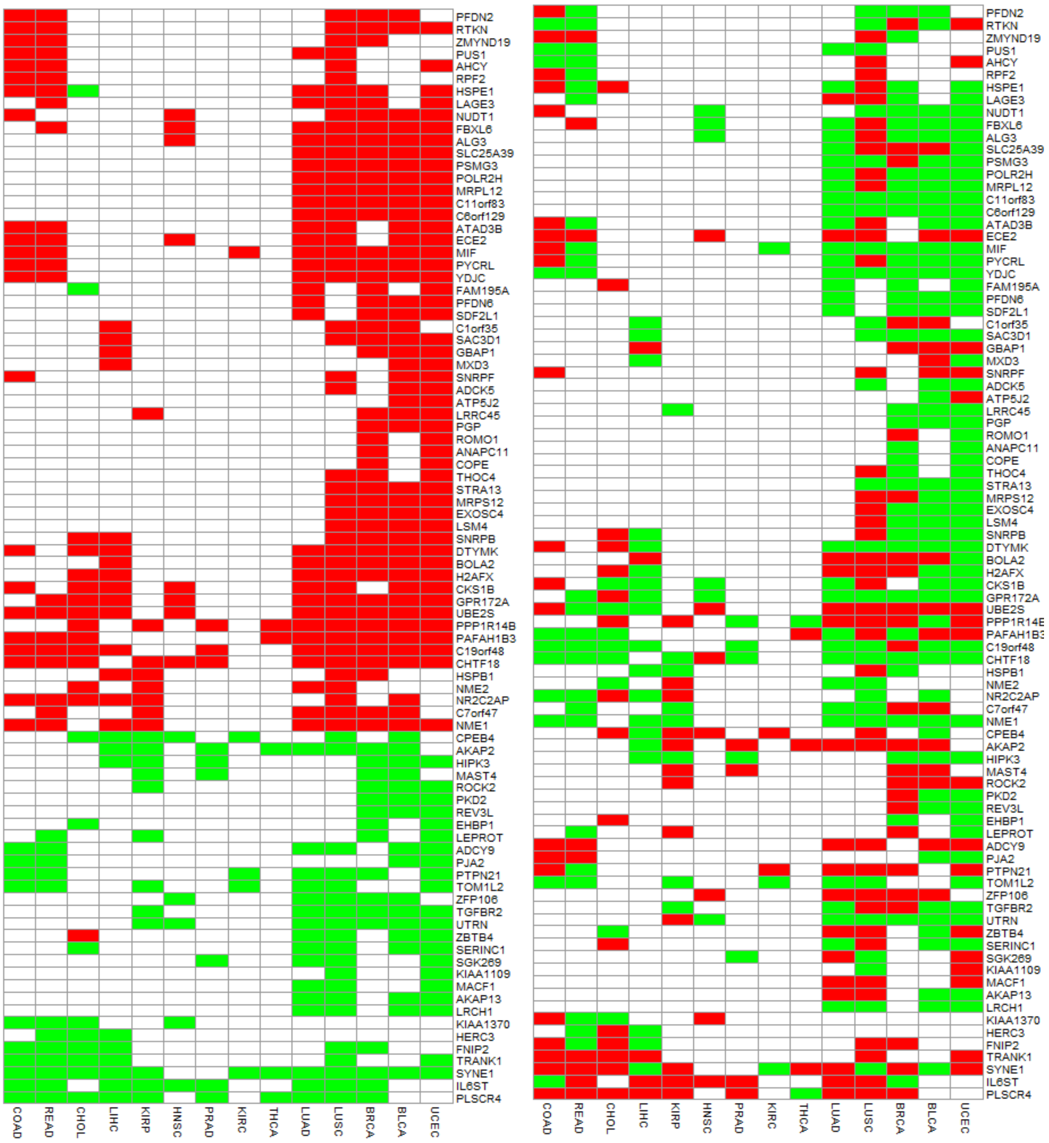

Figure S2. (A) Genes in subnetwork M3 are regulated by many motifs. Each row represents a gene and each column represents a motif. The motif specific transcriptional factors are marked along the columns. If a motif is enriched in the promoter region of a gene, the pix is filling in steelblue. Only significant motifs determined by gProfiler are shown [4]. (B) Gene expression and DNA methylation patterns of subnetwork M3. Left: gene expression pattern. Red (1) and green (-1) mean differentially higher and lower expressed compared to normal samples respectively. White (0) means that the gene is not differentially expressed in the cancer. Rows and columns are ordered according to hierarchical clustering (Euclidean distance and average linkage). Right: DNA methylation pattern. Red and green mean higher or lower DNA methylation level compared to the mean of normal samples. Genes and cancers are arranged in the same order as in the left panel. Only the DNA methylation level of differentially expressed genes are shown.

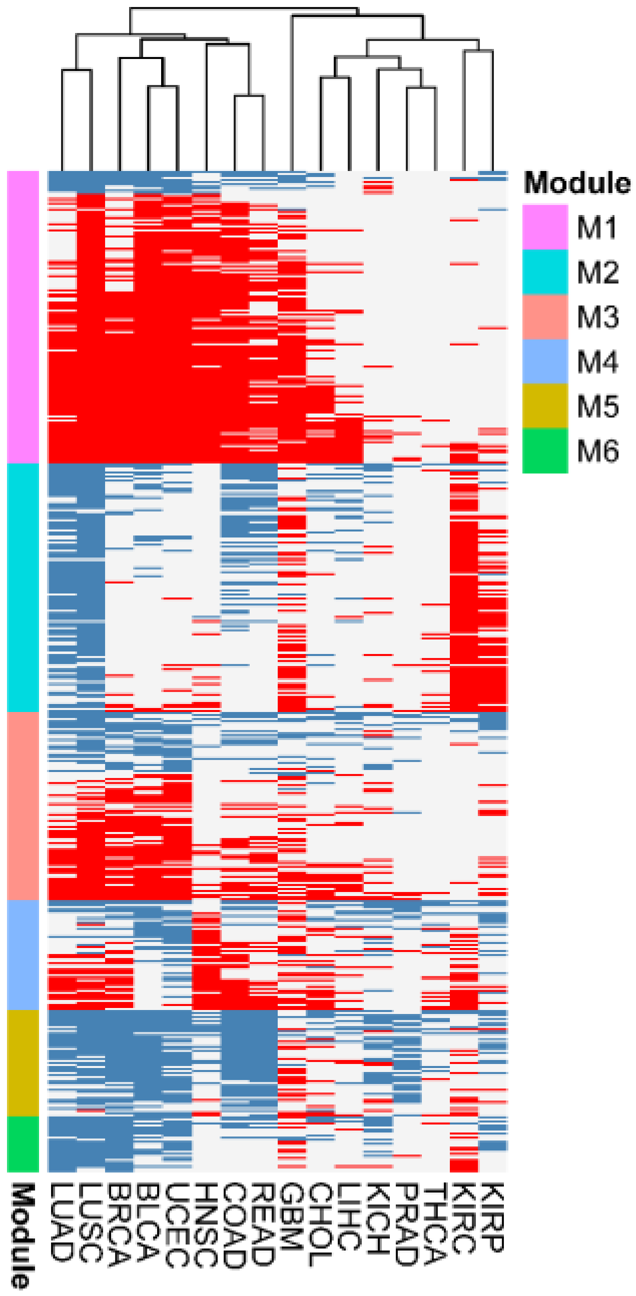

Figure S3. Gene expression patterns of the pan-cancer subnetworks/modules. Rows represents module genes and columns represent cancer types. Red (1) and blue (-1) mean higher and lower differential expression compared to normal samples, respectively. Grey (0) means that the gene is not differentially expressed in the cancer. Cancer types are ordered according to hierarchical clustering (Euclidean distance and complete linkage).

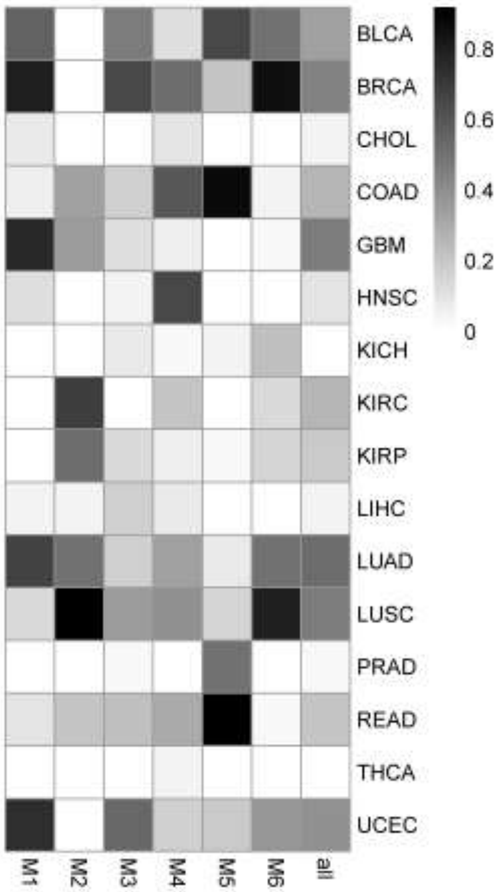

Figure S4. The contributions of each cancer type to the construction of the pan-cancer network. Columns represent the subnetworks M1 to M6 and the whole pan-cancer network (all). Each row correspond to a cancer type. The grey levels are proportioned to the fraction of edges coming from each cancer type in a given network.

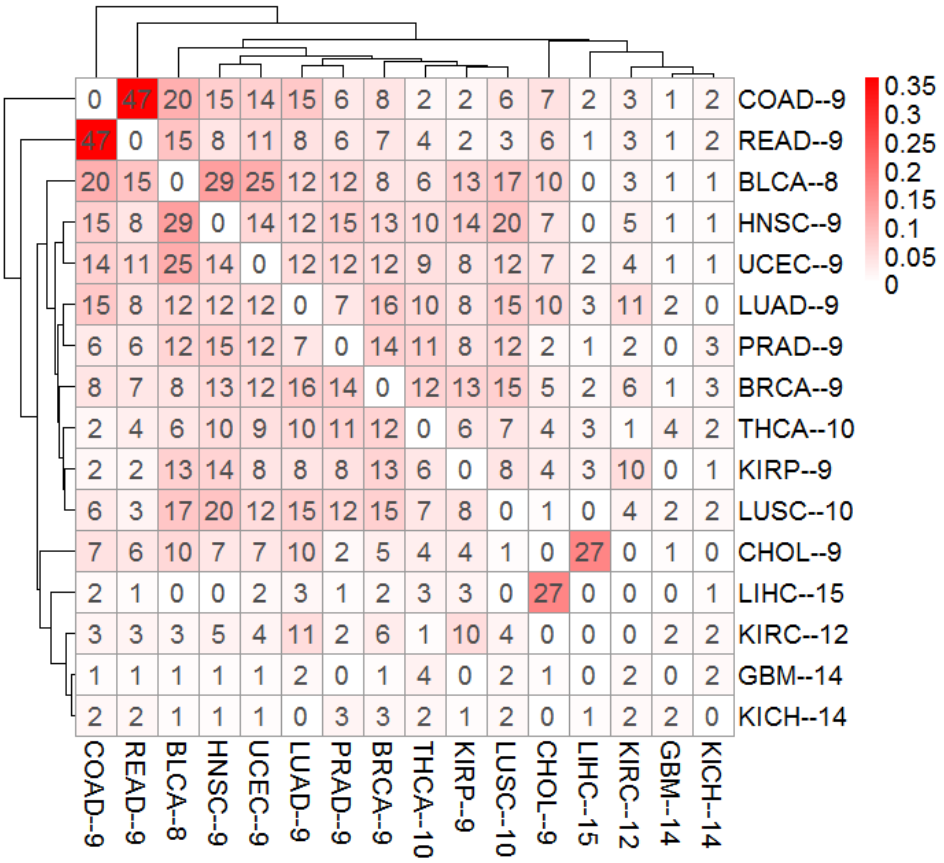

Figure S5. Intersections between cancer type-specific subnetworks. Each row and each column represents a cancer type-specific subnetwork. Colors are proportioned to the Jaccard similarity coefficient of two subnetworks. Numbers of common genes are shown. Rows and columns are ordered according to hierarchical clustering with Euclidean distance and average linkage.

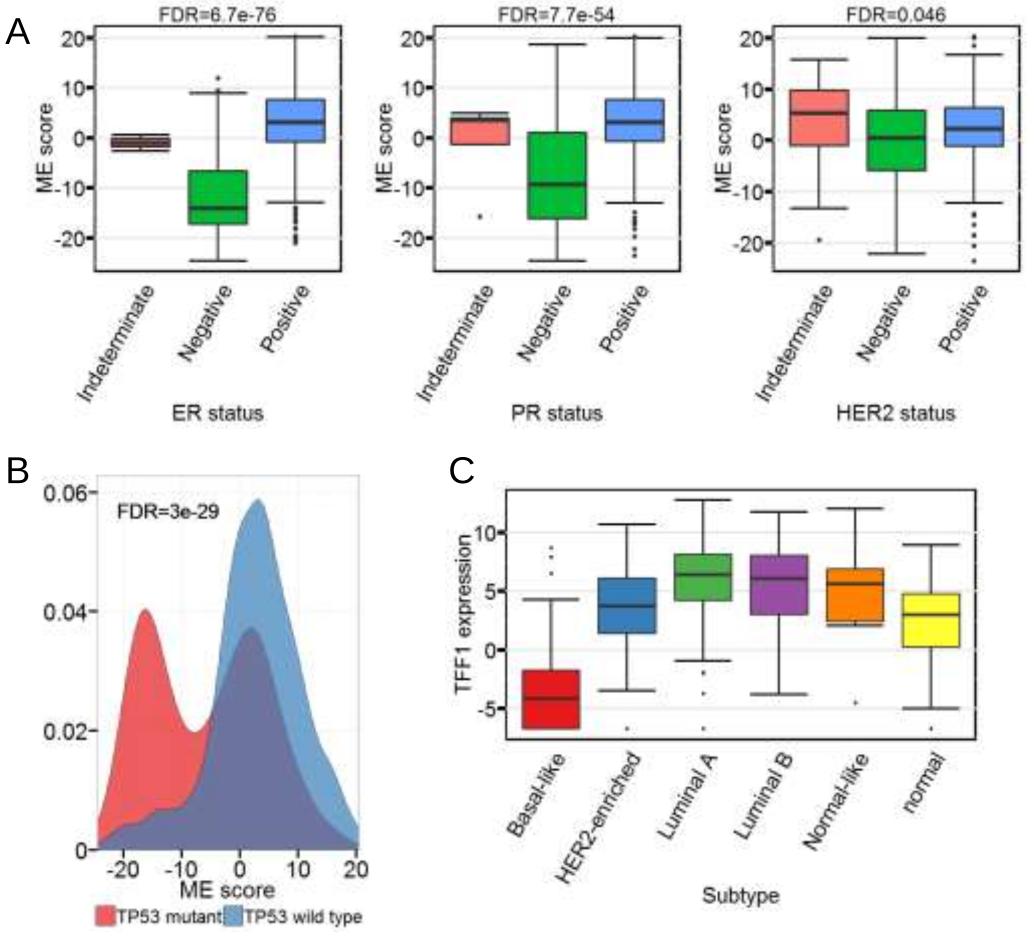

Figure S6. (A) Distribution of ME scores of the BRCA subnetwork in terms of ER, PR and HER2 status. (B) Distribution of ME scores of the BRCA subnetwork in terms of *TP53* mutation status. (C) *TFF1* gene expression distributions (TMM normalized data) in terms of PAM50 subtypes. For box plots, the bottom, top, and middle bands of the boxes indicate the 25th, 75th, and 50th percentiles, respectively. Whiskers extend to the most extreme data points no more than 1.5 interquartile ranging from the box. *FDR* values (or *p* value) for the Kruskal-Wallis test are provided at the top of the boxplots.

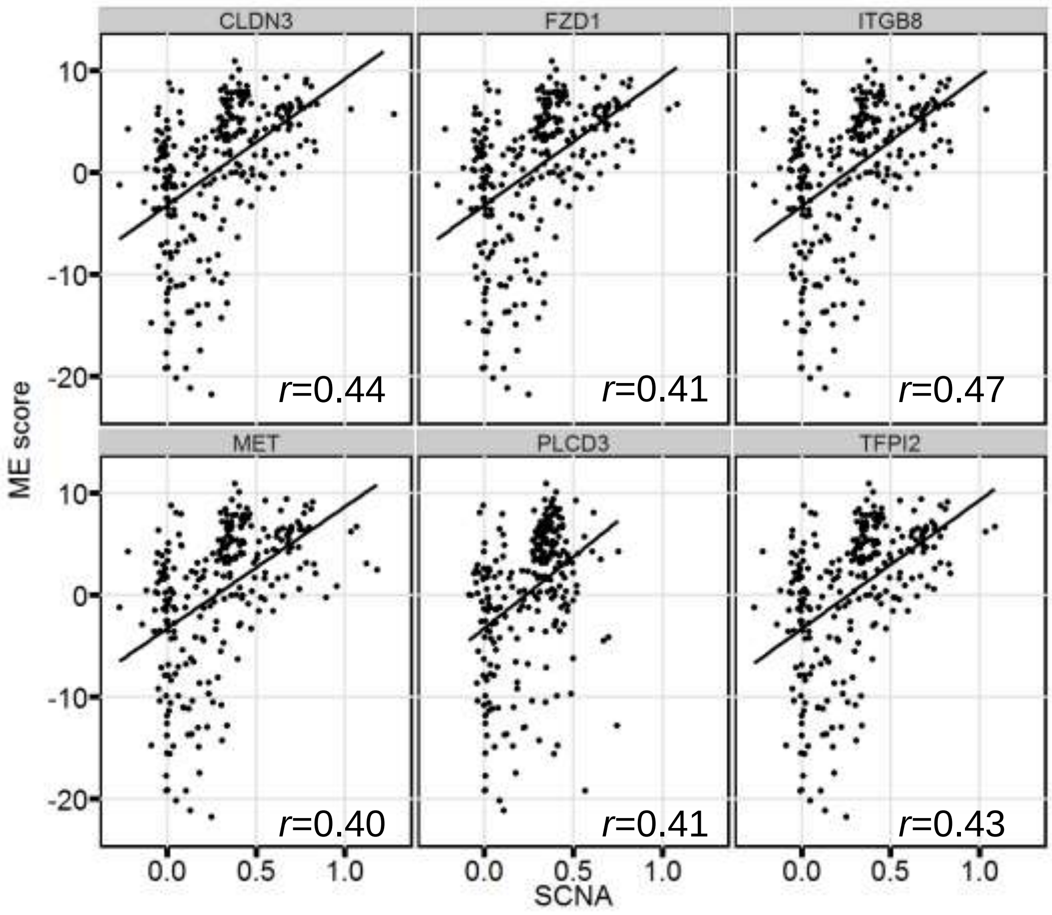

Figure S7. Correlation between the KIRP subnetwork and SCNAs. Scatter plot of *CLDN3*, *FZD1*, *ITGB8*, *MET*, *PLCD3*, *TFPI2* SCNA and ME scores. The regression line is calculated by least squares and shown in the panel. Pearson's correlation coefficients are shown at the bottom of each panel.

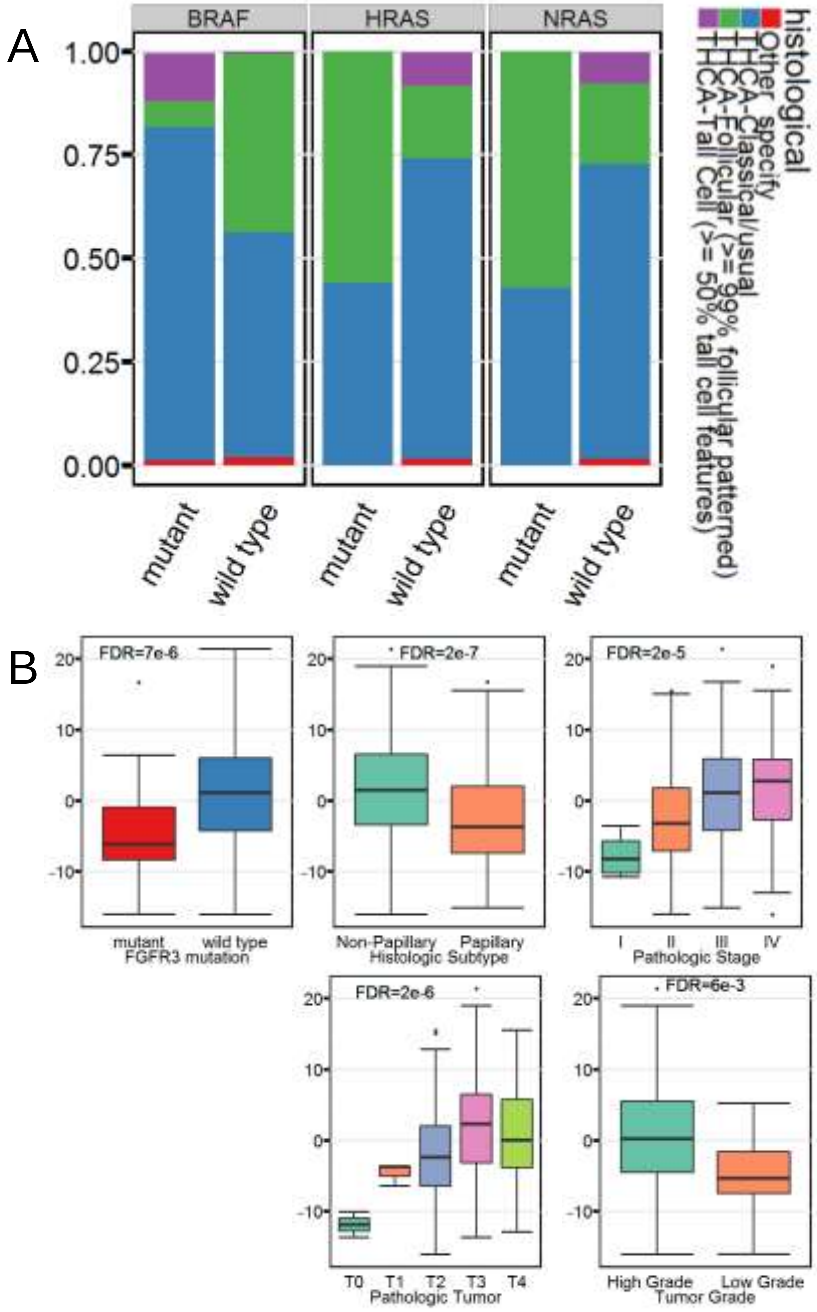

Figure S8. (A) The association between histological diagnosis and *RAF-RAS* mutation status. Different colors indicate diverse histological diagnosis. Rows are the mutation status of *BRAF*, *HRAS* and *NRAS* genes. Columns are the proportion of patients of different histological subtypes. (B) ME scores of the BLCA subnetwork in terms of *FGFR3* mutation status, histologic subtype, pathologic stage, T stage and tumor grade. For box plots, the bottom, top, and middle bands of the boxes indicate the 25th, 75th, and 50th percentiles, respectively. Whiskers extend to the most extreme data points no more than 1.5 interquartile ranging from the box. *FDR* values (or *p* value) for the Kruskal-Wallis test are provided at the top of the boxplots.

A

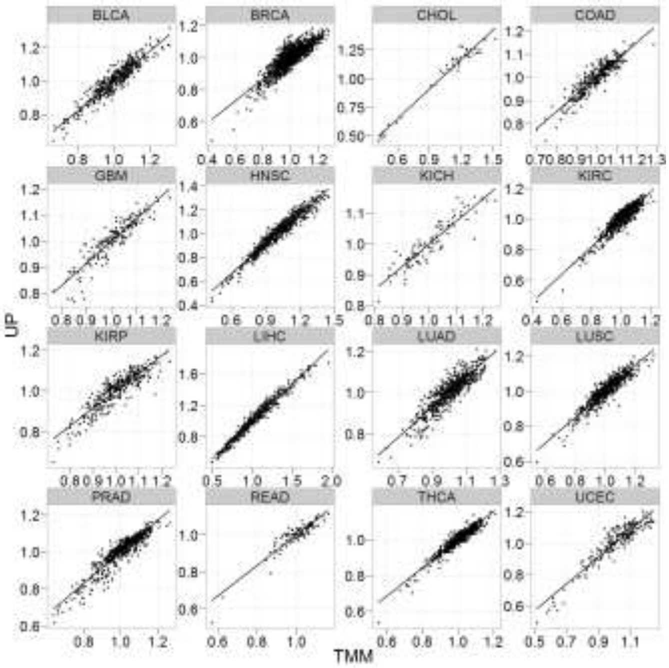

B

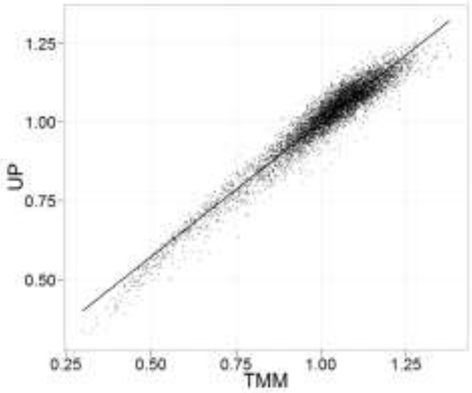

Figure S9. Comparison between the normalization factor of trimmed mean of M-value (TMM) and upper quartile (UQ) normalization methods. X-axis is the normalization factor calculated by TMM, Y-axis is that of UQ.

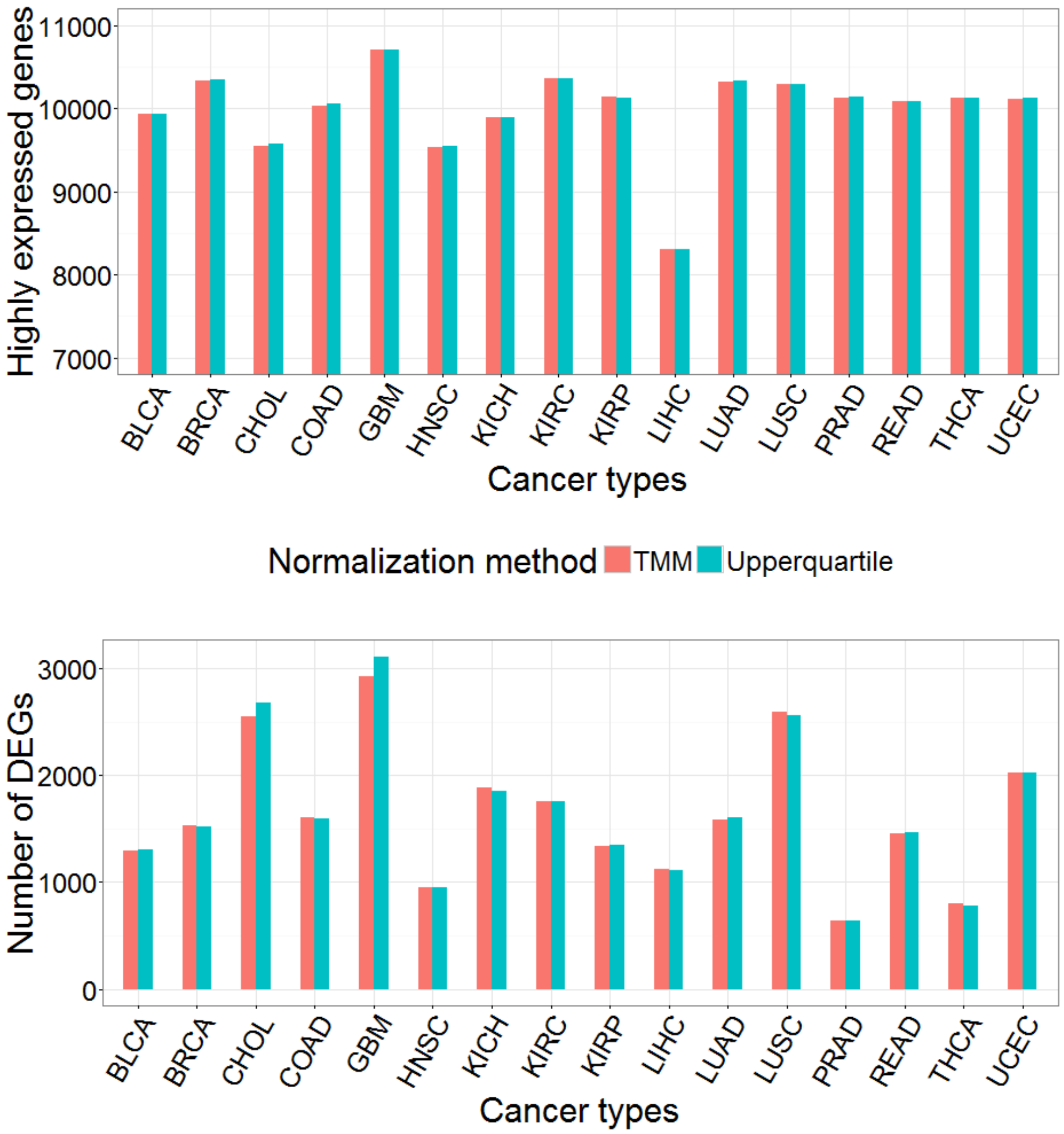

Figure S10. Comparison between TMM and UQ for differentially expression analysis. After normalization, highly expressed genes (more than 50% of samples have CPM≥10) are counted. Under different normalization methods, number of differentially expressed genes (fold change≥2 and FDR≤0.001) are also counted.

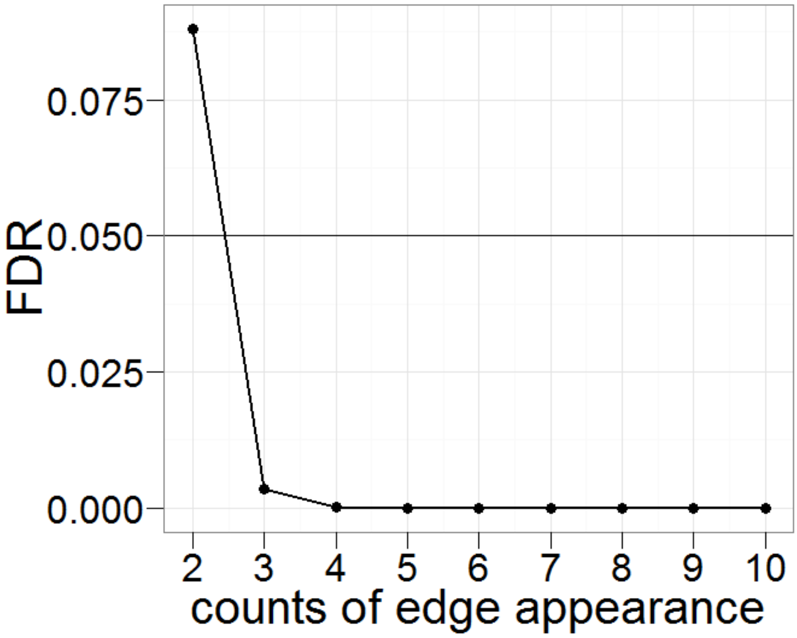

Figure S11. False discovery rate of high-frequency appearances of edges. Given a parameter ***n*** (***n***=2,3,…,14,15,16), FDR values are defined as mean of edges which shows up in ***n*** pseudo networks divided by that of real networks. There doesn't exist any edge which appears in more than 10 pseudo or real networks.

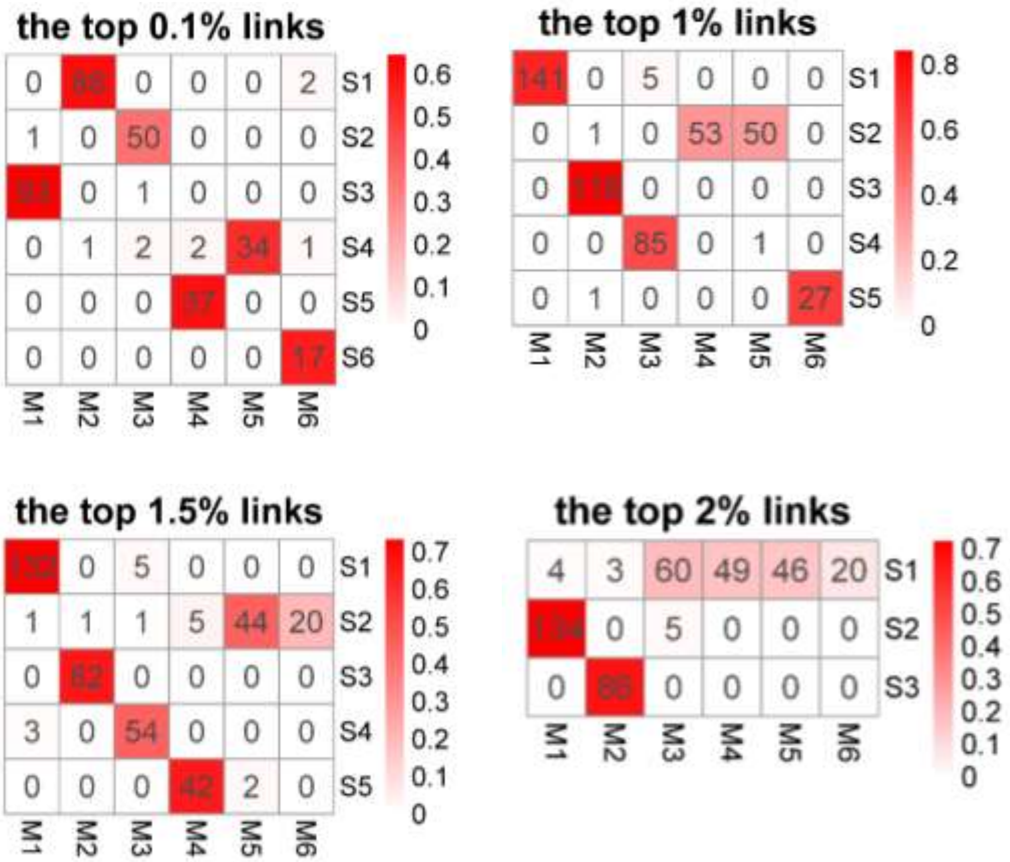

Figure S12. Stability of the pan-cancer subnetworks. Rows correspond to pan-cancer subnetworks (0.5% links and cutoff 3). Columns are subnetworks calculated under other different parameters (0.1%, 1%, 1.5%, 2% links and 2, 3, 4, 4 cutoffs, respectively). Colors are proportioned to Jaccard similarity coefficient of two subnetworks. Numbers of common genes are shown.

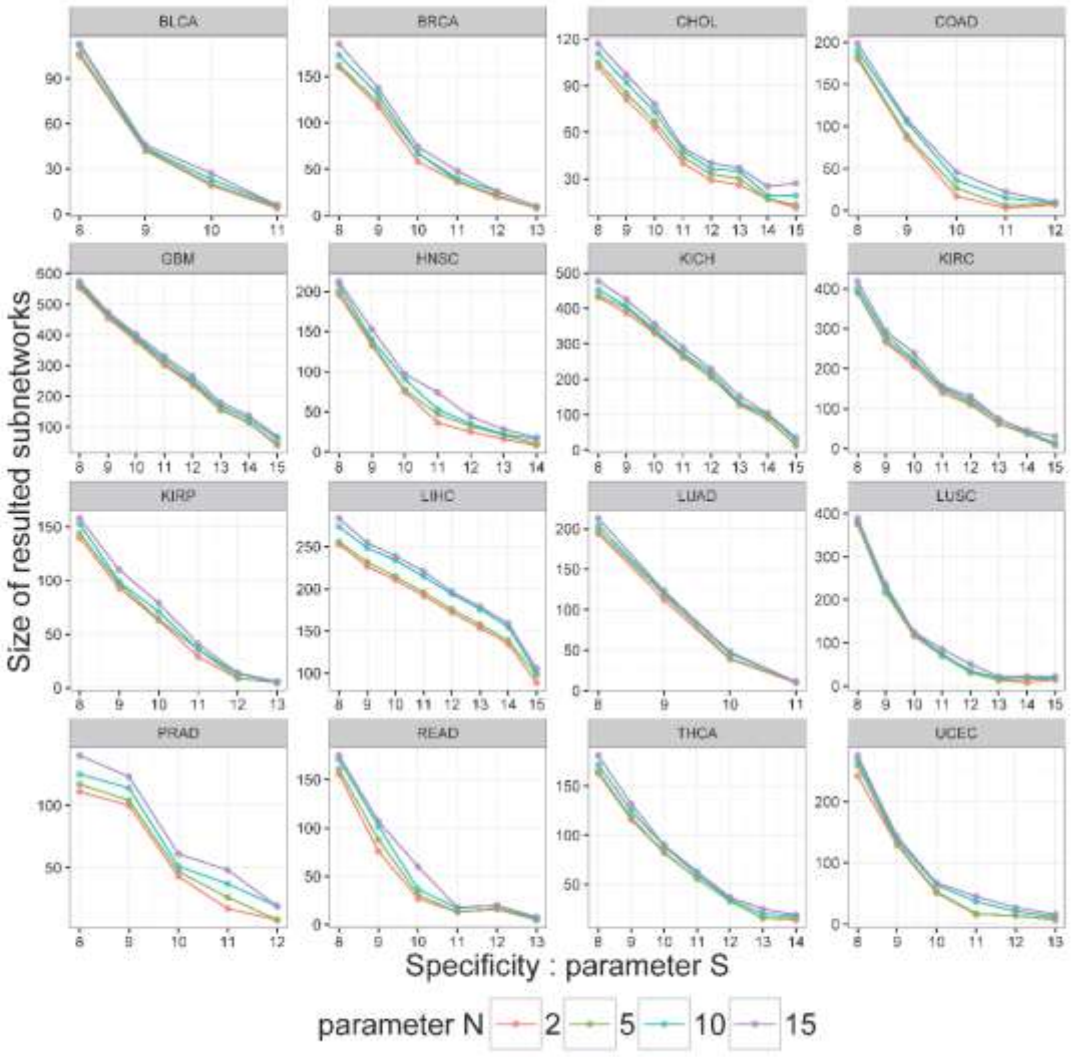

Figure S13. Sizes of cancer type-specific subnetworks under different ***N*** and ***S***. Different colors indicate different ***N***.

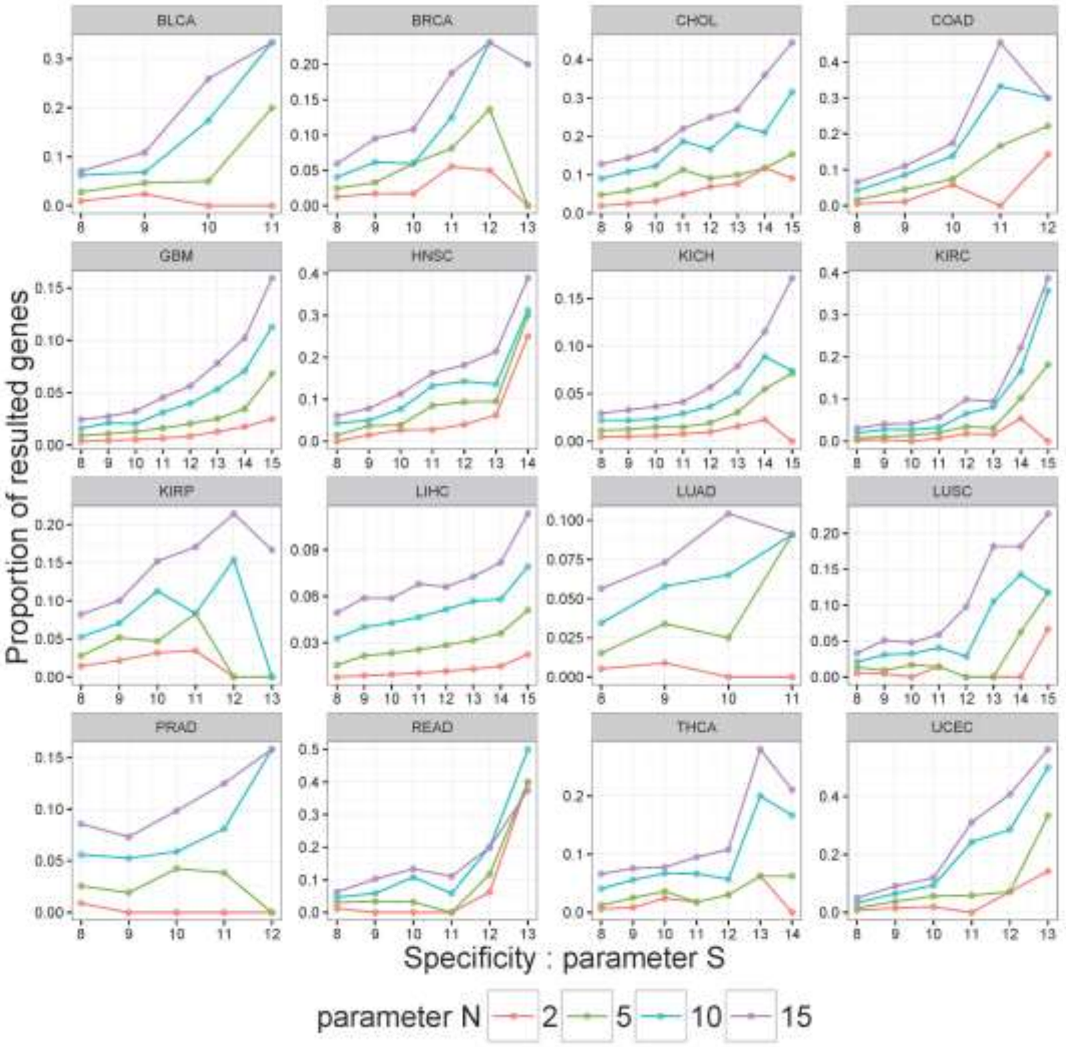

Figure S14. Proportion of the resulted genes in cancer type-specific subnetworks under different ***N*** and ***S***. 'Query genes' are our input genes and 'resulted genes' are those predicted by geneMania. Different colors indicate different ***N***.

References

1. Newman ME: **Finding community structure in networks using the eigenvectors of matrices.** *Phys Rev E Stat Nonlin Soft Matter Phys* 2006, **74:**036104.
2. Mostafavi S, Ray D, Warde-Farley D, Grouios C, Morris Q: **GeneMANIA: a real-time multiple association network integration algorithm for predicting gene function.** *Genome Biol* 2008, **9 Suppl 1:**S4.
3. Benjamini Y, Hochberg Y: **Controlling the False Discovery Rate - a Practical and Powerful Approach to Multiple Testing.** *Journal of the Royal Statistical Society Series B-Methodological* 1995, **57:**289-300.
4. Reimand J, Arak T, Vilo J: **g:Profiler--a web server for functional interpretation of gene lists (2011 update).** *Nucleic Acids Res* 2011, **39:**W307-315.
